# Manifold Learning for Human Population Structure Studies

**DOI:** 10.1371/journal.pone.0029901

**Published:** 2012-01-17

**Authors:** Hoicheong Siu, Li Jin, Momiao Xiong

**Affiliations:** 1 MOE Key Laboratory of Contemporary Anthropology, School of Life Sciences, Fudan University, Shanghai, China; 2 Human Genetics Center, University of Texas School of Public Health, Houston, Texas, United States of America; University of North Carolina, United States of America

## Abstract

The dimension of the population genetics data produced by next-generation sequencing platforms is extremely high. However, the “intrinsic dimensionality” of sequence data, which determines the structure of populations, is much lower. This motivates us to use locally linear embedding (LLE) which projects high dimensional genomic data into low dimensional, neighborhood preserving embedding, as a general framework for population structure and historical inference. To facilitate application of the LLE to population genetic analysis, we systematically investigate several important properties of the LLE and reveal the connection between the LLE and principal component analysis (PCA). Identifying a set of markers and genomic regions which could be used for population structure analysis will provide invaluable information for population genetics and association studies. In addition to identifying the LLE-correlated or PCA-correlated structure informative marker, we have developed a new statistic that integrates genomic information content in a genomic region for collectively studying its association with the population structure and LASSO algorithm to search such regions across the genomes. We applied the developed methodologies to a low coverage pilot dataset in the 1000 Genomes Project and a PHASE III Mexico dataset of the HapMap. We observed that 25.1%, 44.9% and 21.4% of the common variants and 89.2%, 92.4% and 75.1% of the rare variants were the LLE-correlated markers in CEU, YRI and ASI, respectively. This showed that rare variants, which are often private to specific populations, have much higher power to identify population substructure than common variants. The preliminary results demonstrated that next generation sequencing offers a rich resources and LLE provide a powerful tool for population structure analysis.

## Introduction

Next-generation sequencing technologies detect millions or even ten millions of the genomic variants including both common and rare variants [1]–[3]. The structure and organization of the human genome can be completely identified by next-generation sequencing. The fully sequenced population samples of the human genome are a rich resource for uncovering the distribution of genetic variability among populations, identifying the population structure and inferring the demographic histories of natural populations [4], [17]. Next-generation sequencing will broaden the range of questions open to empirical investigation of population genetics and offer unprecedented opportunities for population genetic studies, but also raises great challenges for our data analysis. The next generation sequencing technologies suffer from three limitations: sequence errors, assembly errors, and missing data. The dimension of the population genetics data produced by next-generation sequencing platforms is extremely high. However, the “intrinsic dimensionality” of sequence data which determines the structure of populations is much lower. It is increasingly recognized that the high dimension reduction that can reduce the noises and effectively extract useful population genetics information from the data holds a key to the success of population structure and historical inference from next-generation sequencing data [4]. This motivates us to use dimension reduction techniques to represent high dimensional sequence data in a low dimensional space for population structure studies.

A widely used method for dimension reduction in population genetic studies is principal component analysis (PCA) which searches directions with maximum variability [5]–[9]. PCA is a traditional nonparametric linear data reduction method and is designed to model linear variability in high-dimensional space and applied to inferring axes of human genome variation PCA identifies axes that are uncorrelated and represent maximal variance of the data in a linear projection [10]. The computational simplicity of PCA makes it a major tool for population structure and historical inference from large data sets. However, PCA also suffers from some limitations. It is still difficult to completely reveal substructure of heterogeneous samples collected from continents using PCA [7]. More seriously, PCA is sensitive to data noises and missing values [11].

In the past decade, there has been increasing interest in developing algorithms for recovering compact, informative low dimensional structure hidden in the high dimensional space. The underlying hidden structure of the observed data often arises from a low dimensional manifold embedded in the high dimensional ambient space [11]–[20]. This approach for recovering hidden manifold structure is usually referred to as manifold learning which seeks to design a low-dimensional graph embedding incorporating neighborhood information of the data set [12], [16]. Manifold learning has several remarkable features. First, it develops a low dimensional graph representation of the high dimensional data set which optimally preserves local topological and structural (or neighborhood) information of the original dataset. The structure of the dataset can be characterized by the geodesic distance in isometric mapping (ISOMAP) [13], or geometrical distance between data points in locally linear embedding (LLE) [21], or the Laplacian function of the data points in the Laplacian Eigenmap [12]. In population genetic studies, the geometrical distance between individuals can be measured by their sequence (or genetic) similarity. The neighborhood information among the data points reflects the population structure of the individuals. Second, the manifold learning is a nonlinear data reduction technique. Unlike PCA that searches for representation to maximize the linear variability of the dataset, the manifold learning searches for a nonlinear low dimensional representation which optimally preserves the local neighborhood structure of the data. Third, the neighborhood preserving projection of manifold learning makes it relatively insensitive to outliers, sequencing errors and noises. Outliers in the classical PCA will dramatically increase dimensions to separate outliers from the bulk of the remaining data.

The purposes of this report are twofold. The first is to use LLE [21] as a general framework for inferring relationships among individuals and population structure from next-generation sequencing data in the presence of common and rare variants. The second is to use LLE and LASSO [22] as tools for developing algorithms to identify structure informative SNPs and genomic regions. To achieve this, we first introduce geometric distance and frequency-weighted genetic distance. Then, we use the LLE method as a tool to develop a low dimensional representation of the high dimensional genomic data while preserving the structural relations among the data points. To facilitate application of the LLE to population genetic analysis, we systematically investigate several important properties of the LLE for population structure and history inference and reveal the connection between the LLE and PCA. To achieve the second goal, in addition to analyzing a set of markers significantly correlated with top principal components (PCs) and genomic regions enriched with these PC correlated markers, we develop statistical methods for identifying markers that are significantly correlated with low dimensional representation of the high dimensional genomic data. To further reduce the impact of the sequence errors on the population structure inference and increase the power to identify structure informative genomic regions, we propose to integrate genomic information content in a genomic region and use LASSO regression for collectively testing association of the genomic region with the low dimensional representation of the high dimensional genomic data. Finally, to assess the challenges which the LLE method faces in analyzing next-generation sequencing data, we apply the LLE and PCA to low coverage pilot dataset in the 1000 Genomes Project which was released in July, 2010 [23]. To further evaluate the performance of the LLE for population structure inference, we also apply the LLE and PCA to the PHASE III Mexico dataset of the HapMap released in May, 2010 (ftp://ftp.ncbi.nih.gov/hapmap/genotypes/2010-05_phaseIII) [24]. Our results show that the LLE provide a valuable tool for population structure analysis of next-generation sequencing data.

## Results

### Neighborhood Structure of Genomic Data

**Figure 1:**
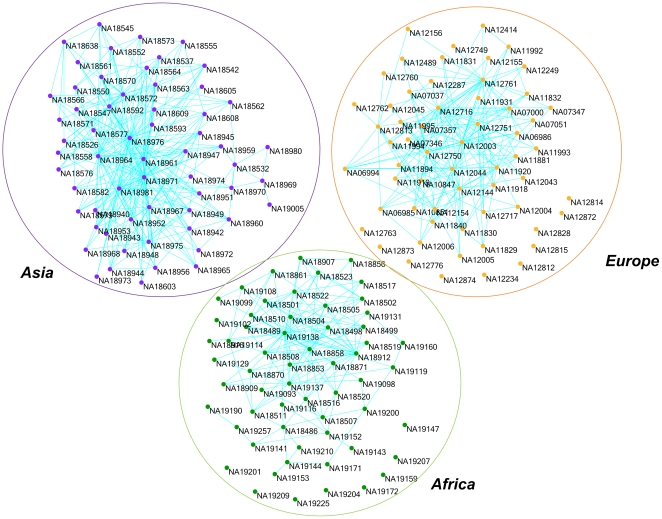
The reconstructed graphs of 179 human genomes from three populations: YRI, CEU and ASI by the LLE algorithms assuming 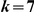 nearest neighbors. Each node in the graph represents an individual. A pair of nodes associated with the non zero weight is connected.

A remarkable feature of the LLE for nonlinear dimensionality reduction is to compute a low dimensional coordinates of high dimensional genomic data by incorporating neighborhood information of the genomic data set. To examine the neighborhood structure of the genomic data, the LLE was applied to the low coverage pilot dataset in the 1000 Genomes Project which were released in July, 2010 (ftp://ftp.1000genomes.ebi.ac.uk). The dataset included 179 unrelated individuals from four populations: Yoruba in Ibadan, Nigeria (YRI, 59 individuals); Utah residents with ancestry from Northern and Western Europe (CEU, 60 individuals); Han Chinese in Beijing, China (CHB, 30 individuals) and Japanese in Tokyo, Japan (JPT, 30 individuals). In this study, CHB and JPT populations were combined as one population (ASI). These samples were sequenced on an average cover rate of 4×. A total of 14,397,437 SNPs on the 22 autosomes were identified. The LLE algorithm first used geometric distance to calculate 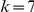 nearest neighbors and computed the weights that best reconstructed each data point from its neighbors. Figure 1 shows a graphical representation of 179 individuals based on 14,397,437 SNPs where each node in the graph represents an individual and each edge represents the connection between a pair of nodes. If a weight associated with a pair of nodes was not equal to zero, two nodes were then connected in the graph. Several remarkable features emerge from Figure 1. First, Figure 1 shows that individuals from YRI, CEU and ASI formed three disconnected subgraphs. It clearly uncovered population structure in the genomic dataset. Second, each subgraph has several hubs with high node degrees. These individuals might play an important role in the evolution of population. Third, several isolated individuals in the YRI and CEU subgraphs were observed. These individuals shared less genetic similarity with other individuals in the populations.

**Figure 2:**
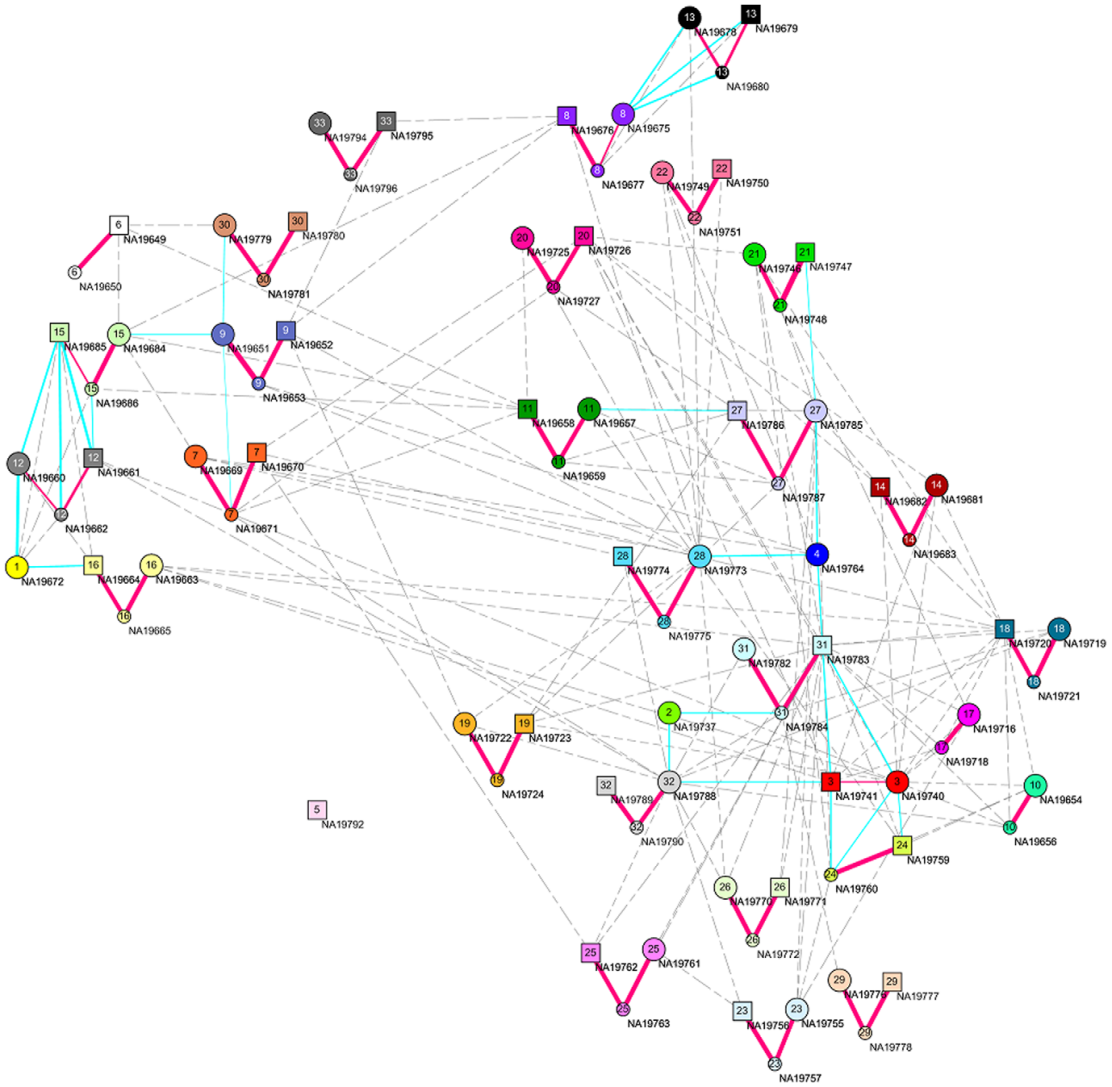
The reconstructed graph of Mexican Americans in the Los Angeles, California (MEX) population data of HapMap phase III released in May 2010 with 86 individuals from 33 families. A Father is represented with a large size square, a mother is represented with a large size circle and a child is represented with a small size circle. The number insight the squares and circles denotes the index of the family. The strong connections between individuals are represented by real lines and the weak connections between individuals is represented by dotted lines.

It can be shown that the weights in the LLE are a function of kinship coefficients or the probability sharing IBD (identical by descent) among individuals (See Material and Methods). Therefore, the LLE algorithms can be used to estimate the genetic relationships between individuals. To further illustrate the power of the LLE for discovering the genetic relations among individual in the dataset, the LLE was applied to the PHASE III Mexico dataset of the HapMap released in May, 2010, which included 86 individuals from 33 families (ftp://ftp.ncbi.nih.gov/hapmap/genotypes/2010-05_phaseIII). These samples were genotyped on both the Illumina Human 1 M BeadChip and the Affymetrix Genome-Wide Human SNP Array 6.0 platforms. After quality control and removing SNPs with missing genotype, the final data set for our analyses consisted of 374,434 SNPs on the autosomes. Similar to Figure 1, Figure 2 presents the graph of 86 individuals computed by the LLE algorithms where strong connections between individuals were represented by real lines with the width of the lines denoting the degree of connection and weak connections were represented by dotted lines. Figure 2 demonstrates that the LLE can reconstruct 33 families. We observe that all parents and their children were connected by thick real lines. It is surprising that all family structures discovered by the LLE are completely consistent with the records in the original dataset. Except for family 5 where only the father was genotyped, all remaining 32 families were connected. Families 8 and 13 and families 12 and 15 were strongly connected.

**Figure 3:**
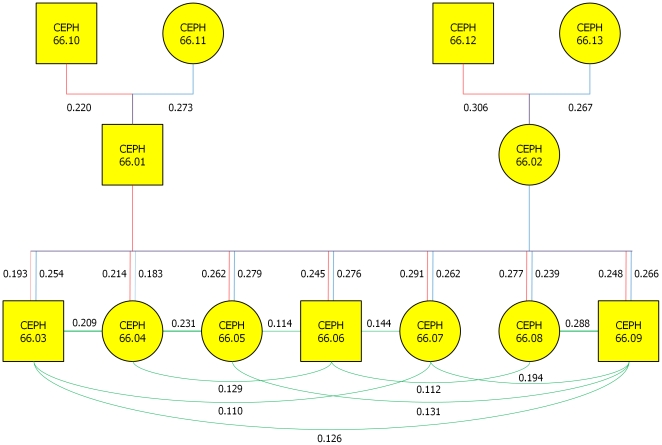
A reconstructed three generation French family from the CEPH dataset by the LLE where a rectangle represents a male and a circle represents a female, assuming *k* = 7. The numbers next to each edge are the weights that reconstruct each of the data points from its neighbors in the LLE. The red real lines denote connections between the grandfathers and their children or between fathers and their children. The blue real lines denote connections between the grandmother and their children or between the mothers and their children. The green lines denote the connections between the grandchildren.

Next we consider three generation families with much less genotyped SNPs. Figure 3 plots a reconstructed three generation French family from the CEPH dataset by the LLE where 765 SNPs were genotyped. We observed that the estimated pedigree structures by the LLE algorithms were consistent with the reported pedigree structures very well (http://ccr.coriell.org).

### Block Structure and Ancestral Representation of Populations in Low Dimensional Space

**Figure 4:**
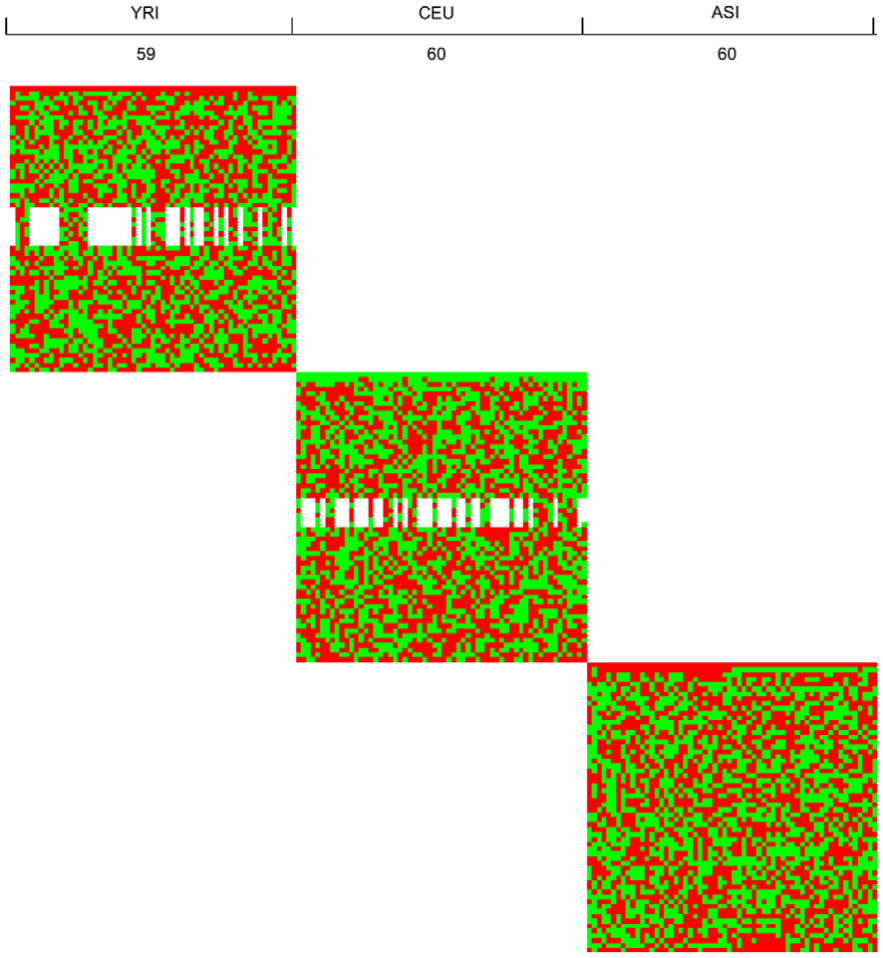
Low-dimensional coordinates in a non-linear dimensional projection of all 14,397,437 SNPs of 179 individuals from three populations mapped by the LLE where the X axis is the individual index and the Y axis is the eigenvalue arranged from large to small. The color of the pixel at (X, Y) is the value of the eigenvector corresponding to eigenvalue at Y for individual X . Green color represents negative values, red color represents positive values and white color represents values close to zero.

To evaluate the topology (genetic structure) preservation of the LLE for mapping high dimensional genomic data to low dimensional space, we plot Figure 4 to visualize the map of all 14,397,437 SNPs of 179 individuals from three populations to low dimensional space by the LLE where the X axis is the individual index and the Y axis is the eigenvalue that is arranged from large to small. The color of the pixel at (X, Y) is the value of the eigenvector corresponding to eigenvalue at Y for individual X. Green color represents negative values, red color represents positive values and white color represents values close to zero. Figure 4 clearly shows that the LLE algorithm mapped individuals from three populations to three separate clusters in low dimensional embedding. It is interesting to observe that eigenvectors (low dimensional coordinates of the genomic data) have remarkable block structure. Each block represents a specific population.

**Figure 5:**
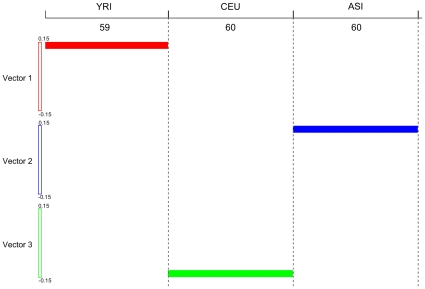
Three eigenvectors in the eigenspace of zero eigenvalue for the nonlinear dimensional mapping of all 14,397,437 SNPs of 179 individuals from three populations mapped by the LLE define axes of populations: YRI, CEU and ASI. Three intervals along the y-axis ranging from −0.15 to 0.15 were used to represent the values of the components in the eigenvectors (low dimensional representations) and the x-axis represents the individual index. The red color represents the YRI samples, green color represents the CEU samples and blue color represents the ASI samples.

If several populations are completely separated, the eigenvectors with zero eigenvalue can be used to define the axes of population ancestry. The LLE for mapping the low coverage pilot genomic data in the 1000 Genomes Project to the low dimensional data space found four eigenvectors with zero eigenvalue. The unit eigenvector with all equal components should be removed to enforce the constraint: 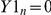. The remaining three eigenvectors with zero eigenvalue were coordinates in low dimensional space of the high dimensional genomic data of 179 samples from three populations. Figure 5 shows that these three eigenvectors define the axes of the populations: YRI, CEU and ASI. We can link each population to an eigenvector. Three eigenvectors can form three axes and an axis corresponds to a population. The LLE projects samples from three populations onto three orthogonal axes with samples from a population projected onto its corresponding axis.

**Figure 6:**
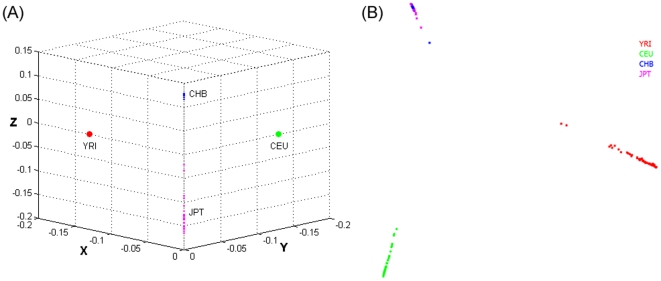
**A.** Three eigenvectors in the eigenspace of zero eigenvalue for the nonlinear dimensional mapping of all 14,397,437 SNPs of 179 individuals from four populations YRI, CEU, CHB and JPT mapped by the LLE using the allele frequency weighted genetic distance. The corresponding coordinate of the eigenvector associated with individuals from YRI, CEU, and CHB and JPT (ASI) was mapped to the x axis, the y axis and z axis, respectively. **B.** Graphic representation of the first two PCs for 179 individuals from four populations YRI, CEU, CHB and JPT on 14,397,437 SNPs.

To assess the impact of different measures of genetic distance between individuals on the performance of LLE for investigating population structure, we plot Figure 6A showing the corresponding coordinate of the eigenvector associated with individuals from YRI, CEU, and CHB and JPT (ASI) mapped by the LLE using the allele frequency weighted genetic distance. The individuals from YRI and CEU were mapped to a point in the X axis and Y axis, respectively, and individuals from the CHB and JPT were mapped to the Z axis. It is clear from Figure 6 that the individuals from the CHB and JPT were completely separated. However, as shown in Appendix S1, the mapped points of the individuals in the Z axis from the CHB and JPT by the Euclidean distance cannot be well separated except for the individuals NA18976 and NA18532. This demonstrates that the genetic distance measures between individuals have a large impact on the performance of the LLE to study the population structure.

To further evaluate the performance of the LLE, we plot the first two PCs for 179 individuals from four populations YRI, CEU, CHB and JPT on 14,397,437 SNPs in Figure 6B to compare the power of the LLE and the popular PCA for detecting the population structure. Unlike in Figure 6A where the individuals from the CHB and JPT can be well separated by the LLE algorithms, Figure 6B shows that the individuals from the CHB and JPT cannot be well separated by the PCA.

The LLE has very nice property that the genetic relationship information in the original genomic data will be preserved in their low dimensional projection. We have shown that the low dimensional coordinates are a function of kinship coefficients of the individuals (Appendix S2). Therefore, we can expect that the weights that are calculated by the original genomic data can be well approximated by the weights that are calculated by the low dimensional coordinates. To confirm this, we plot Appendix S3, that shows the differences between weights estimated by the original genomic data and the low dimensional coordinates. Appendix S3, shows that their differences in weights are very small. However, we can also see from Appendix S3, that the difference between the weights estimated by the original genomic data and PCA are quite large. This further shows that the low dimensional coordinates may include more genetic information than the principal components.

### Fine-Scale Population Structure by the LLE

**Figure 7:**
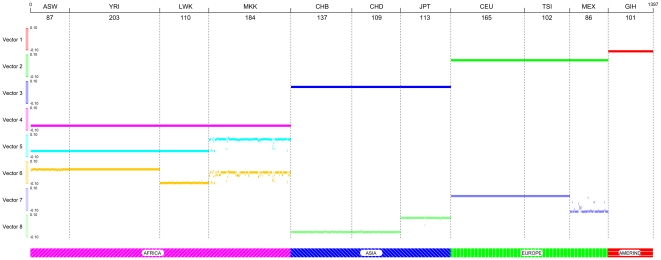
Eight eigenvectors for the nonlinear dimensional mapping of 374,434 SNPs on the autosomes of 1,397 individuals from 11 populations by the LLE where the X axis represents an individual index in each population and the eight eigenvectors (low dimensional representations) were placed along the Y axis.

To investigate the properties of the LLE for discovering fine-scale population structure, the LLE was applied to the PHASE III dataset of the HapMap released in May, 2010 (ftp://ftp.ncbi.nih.gov). This dataset included 1,397 individuals from eleven populations: Yoruba in Ibadan, Nigeria (YRI); Utah residents with ancestry from Northern and Western Europe (CEU); Han Chinese in Beijing, China (CHB) and Japanese in Tokyo, Japan (JPT); Luhya in Webuye, Kenya (LWK); Maasai in Kinyawa, Kenya (MKK); Toscani in Italy (TSI); Gujarati Indians in Houston, Texas (GIH); Chinese in Metropolitan Denver, Colorado (CHD);Mexican Americans in Los Angeles, California (MEX); and African Americans from the Southwestern United States (ASW). The samples were genotyped on both the Illumina Human 1 M BeadChip and the Affymetrix Genome-Wide Human SNP Array 6.0 platforms. After quality control and removing SNPs with missing genotyping, a total of 374,434 SNPs on the autosomes were used for our analysis. The results of the LLE were shown in Figure 7 where the X axis represents an individual index in each population and the eight eigenvectors (low dimensional coordinates) with increasing eigenvalues were placed along the Y axis. Similar to Figure 5, four continental populations: Africa, Europe and Mexican American, Asia and Gujarati Indians were completely separated by the four smallest eigenvectors. Eigenvector 7 can separate Europe and Mexican American populations and eigenvector 8 can well separate Chinese and Japanese populations. Combinations of eigenvectors 5 and 6 can further distinguish the YRI, ASW, LWK and MKK.

### SNPs Significantly Correlated with Low Dimensional Coordinates for Structure Identification

A popular method for detecting structure informative SNPs and genomic regions is to identify SNPs that are significantly correlated with PCs [10], [25]. In this report, we show that SNPs significantly correlated with the low dimensional coordinates (representations) can also be used to measure local contributions to structure inference. To illustrate this, we present Table 1 in which the number of SNPs significantly correlated with top low-dimensional coordinates in a non-linear dimensional projection which define the populations and the PC with the largest eigenvalue is listed. The P-values after Bonferroni correction for multiple tests for declaring significance are 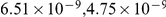
and 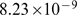
for the samples from the CEU, YRI and ASI, respectively. For the convenience of discussion, the SNPs that are significantly correlated with the low-dimensional coordinates in a non-linear dimensional projection or PC are referred to as ancestry informative SNPs. We observed that 40.3%, 59.1% and 28.6% of the total SNPs in the CEU, YRI and ASI were ancestry informative SNPs, respectively, and YRI had the largest number while ASI had the smallest number of ancestry informative SNPs. It is also interesting to find that the majority of rare variants (89.2%, 92.4% and 75.1% of the rare variants in the CEU, YRI and ASI, respectively) is the ancestry informative SNPs. The rare variants arose recently and are often population specific. The populations with recent expansion have a higher proportion of rare variants. Therefore, rare variants are often likely to be ancestry informative markers [26]. The number of ancestry informative SNPs identified by the LLE algorithms and PCA is close except for ASI where the number of ancestry informative rare SNPs identified by PCA is much smaller than that identified by the LLE algorithms. We also observe that the proportion of shared ancestral informative SNPs identified by LLE and PCA is very high except for ASI.

**Table 1:** Number of SNPs significantly correlated with top low dimensional coordinates and PCs.

Population	CEU	YRI	ASI
Individuals	60	59	60
P-value for significance	6.51E-09	4.75E-09	8.23E-09
All SNPs	7,681,165	10,518,225	6,077,954
LLE Significant	3,099,007	6,216,648	1,740,342
PCA Significant	3,413,850	6,124,608	1,272,679
LLE (%)	40.3%	59.1%	28.6%
PCA (%)	44.4%	58.2%	20.9%
Common SNPs	5,853,886	7,382,215	5,259,537
LLE Significant	1,468,999	3,318,249	1,125,869
PCA Significant	1,860,653	3,266,439	1,172,668
LLE (%)	25.1%	44.9%	21.4%
PCA (%)	31.8%	44.2%	22.3%
Rare SNPs	1,827,279	3,136,010	818,417
LLE Significant	1,630,008	2,898,399	614,473
PCA Significant	1,553,197	2,858,169	100,011
LLE (%)	89.2%	92.4%	75.1%
PCA (%)	85.0%	91.1%	12.2%

Ancestry-informative markers in the LLE and PCA are identified by a simple regression where the low dimensional coordinates and principal component score are regressed on a genomic variant variable. SNPs with P-values less than threshold after Bonferroni correction for multiple tests are selected as LLE or PCA significantly correlated markers.

**Figure 8:**
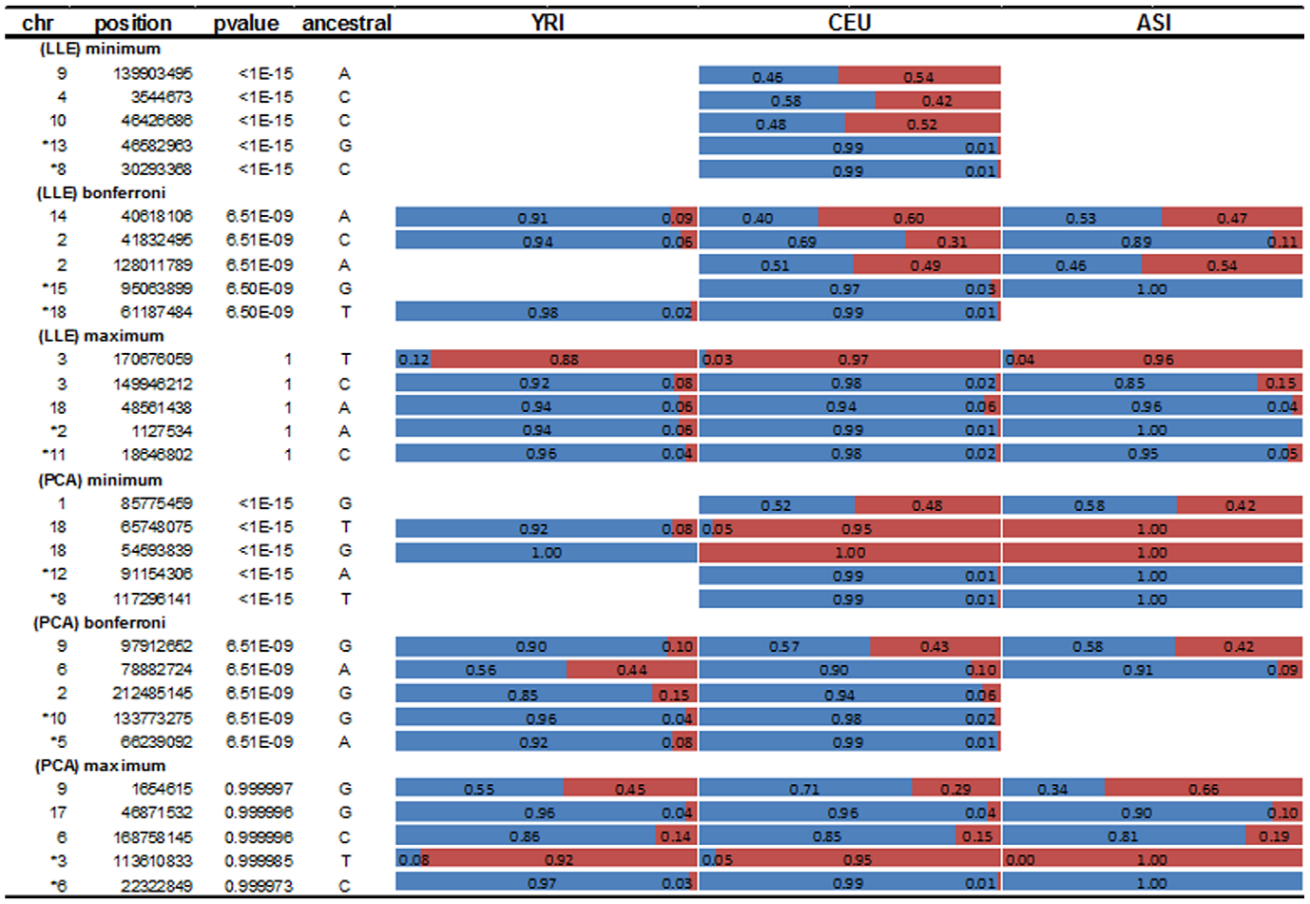
DNA variation pattern of the LLE-correlated, CEU specific SNPs across populations. Raw with star denotes rare alleles (MAF≤5%), raw without star denotes common alleles (MAF>5%); (LLE) minimum or (PCA) minimum indicates that SNPs are selected by the smallest p-value; (LLE) Bonferroni or (PCA) Bonferroni indicates that SNPs are selected by the p-values around the threshold of significance after Bonferroni correction for adjusting multiple testing; (LLE) maximum or (PCA) maximum indicates SNPs are selected by the highest p-value.

Next we study the frequency pattern of the ancestral informative SNPs across populations. Some examples illustrating the frequency pattern of the ancestral informative SNPs for CEU are presented in Figure 8 where the top 3 most informative common and top 2 most informative rare SNPs identified by the LLE algorithms and PCA, respectively are listed. As shown in Figure 8 where the blue color represents an ancestral allele and the red color represents a non-ancestral allele, the ancestry informative SNPs are population specific. These SNPs characterize local changes in ancestry along the genome. For example, the top informative ancestral SNP RS 139903495 identified by the LLE algorithms is a CEU specific SNP. It has an ancestral allele A. The frequency of allele A in the YRI and ASI is 0. In other words, the samples from the YRI and ASI show no polymorphism at RS 139903495. The mutation at this SNP is private for the CEU. However, samples from the CEU showed significant DNA variation at this SNP. The frequency of an ancestral allele was reduced to 0.46. The top informative ancestral rare SNPs identified by the LLE algorithms shared the same feature with the informative ancestral common SNPs. However, both top informative ancestral common and rare SNPs identified by PCA do not show such population specific features. We observe that these SNPs show DNA variations at least in two populations. This observation demonstrates that the LLE algorithms can identify the most informative ancestral SNPs and unravel finer population structure than PCA methods. From Figure 8 we also observe that when the P-values for declaring significance for informative ancestry are increased this remarkable population specific feature for the informative ancestral SNPs identified by the LLE algorithms disappeared. Following this argument, we can expect that un-informative ancestral SNPs will show DNA variations across the three populations (Figure 8). We can observe the similar features for YRI and ASI population specific SNPs (Appendix S4 and Appendix S5).

### Structure Informative Genomic Regions

**Figure 9:**
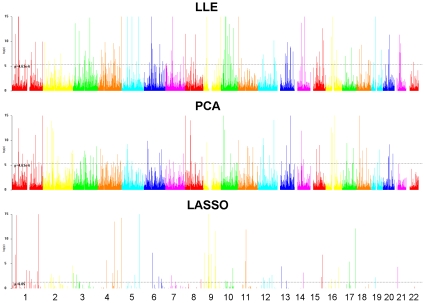
The distribution of structural informative genomic regions identified by the LASSO, LLE and PCA for CEU samples. The genome was divided into nonoverlapping 250 kb bins (*x* axis), and the *y* axis represents the P-value for testing whether the genomic region is significantly structure informative.

The evolutionary forces such as genetic drift, positive natural selection, recombination, and admixture and population bottleneck shape the patterns of population structure. The relative effects of various evolutionary forces on the genomic information contents are difficult to separate. However, we can identify structure informative genomic regions that may have implication in characterizing locus-specific patterns of evolutionary forces. In this report, two approaches are used to identify structure informative genomic regions. One approach is to integrate genomic information content in a genomic region and use the LASSO regression (Materials and Methods) that uses a 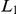
penalty to achieve a sparse solution for identifying structure-informative genomic regions that characterize local changes in ancestry. A second approach is to identify regions significantly enriched with ancestry informative SNPs identified by either the LLE algorithms or PCA (see Material and Methods). To search for the structure informative genomic regions across the genome, we divide the genome into nonoverlapping 250 kb bins. The total number of regions is 10,805. Table 2 lists the number of structure informative genomic regions (P<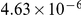) where LLE and PCA represent that we use the Fisher Exact Test for testing whether the region is enriched with LLE-correlated SNPs or PCA-correlated SNPs (P-value<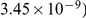 than expected by chance, and LASSO represents a structure information region that is identified by LASSO regression [22]. Table 2 shows that the number of regions identified by the LLE algorithms or PCA is almost sixteen times than that identified by the LASSO method. The number of significant regions shared by the LLE and LASSO method, and the number of significant regions shared by the LLE and PCA is close. They share about half the structure informative regions. However, the number of significant regions shared by three methods: LLE, PCA and LASSO is only 38. The distribution of structural informative genomic regions identified in the CEU samples by the three methods is shown in Figure 9. The distribution of structural informative genomic regions in the YRI and ASI is shown in Appendix S6 and Appendix S7, respectively. Some regions on chromosomes 1 and 4, and HLA regions on chromosome 6 demonstrate high significance in ancestry although in general structural informative regions are widely distributed across the genome, which is consistent with the conclusion of Biswas et al. 2009 [25].

**Table 2:** Number of structure informative genomic regions.

	CEU	YRI	ASI
All Regions	10,805	10,805	10,805
LLE Significant Regions	3,575	4,128	4,039
PCA Significant Regions	3,674	3,674	3,674
LASSO Significant Regions	219	219	228
LLE∩PCA	1,849	3,213	1,984
LLE∩LASSO	87	94	85
PCA∩LASSO	68	90	66
LLE∩PCA∩LASSO	38	81	39

**Figure 10:**
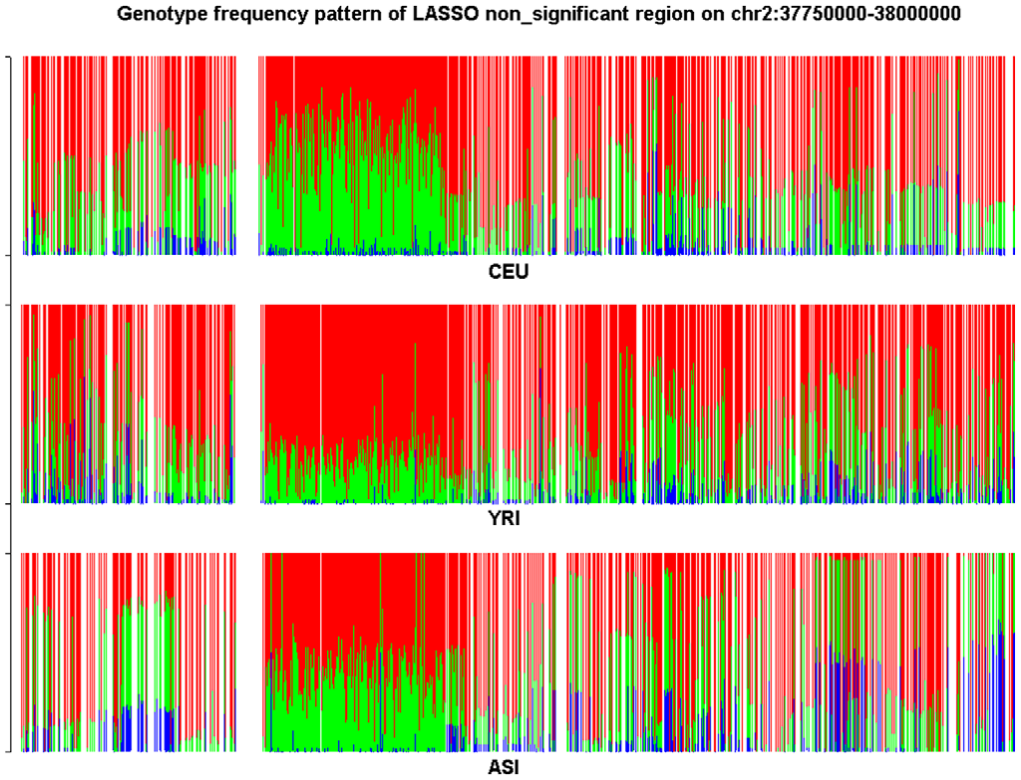
Genotype frequency pattern of the SNPs within the structure significantly informative genome region located on chromosome 2 between 32,750 kb and 32,500 kb which was identified by the LLE and PCA methods, but non-significant by the LASSO method for CEU samples. The red color represents the homozygous genotype with the main allele, green color represents the heterozygous genotype and blue color represents the homozygous genotype with the minor allele.

**Figure 11:**
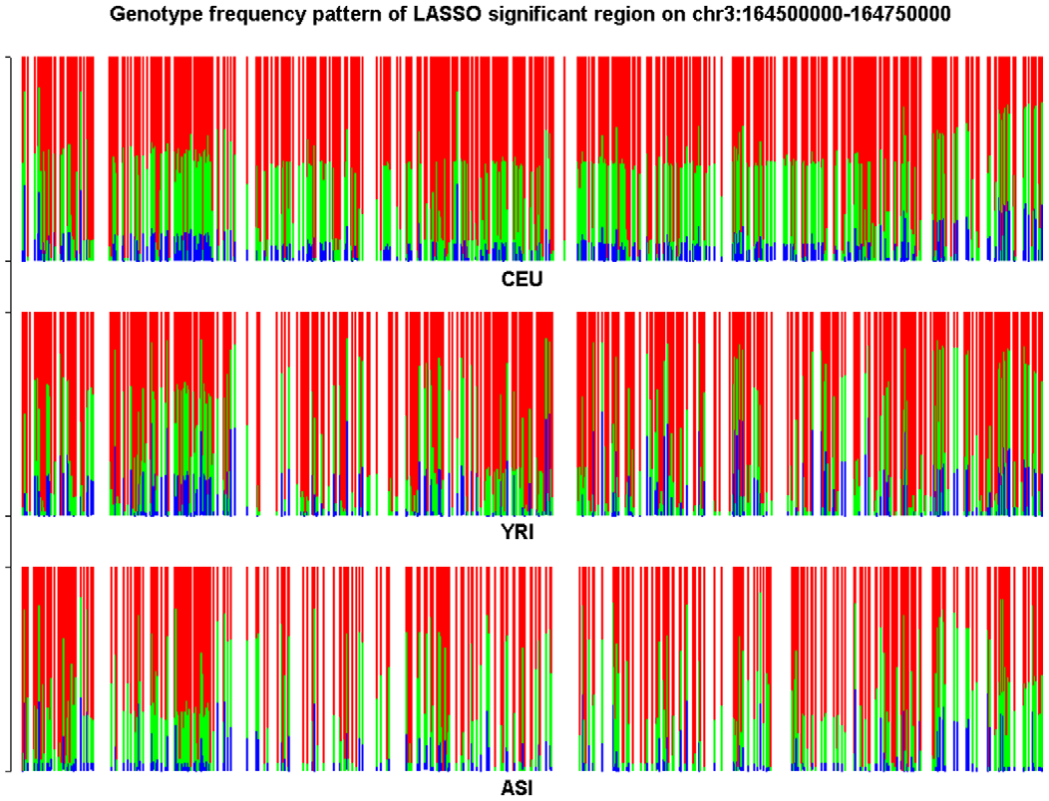
Genotype frequency pattern of the SNPs within the structure significantly informative genome region located on chromosome 3 between 164,500 kb and 164,750 kb which was identified by the LASSO method, but non-significant by the LLE and PCA method for CEU samples. The red color represents the homozygous genotype with the main allele, green color represents the heterozygous genotype and blue color represents the homozygous genotype with the minor allele.

To characterize the features of the structure informative genomic regions and investigate the difference in the genotype frequency pattern between the structure informative genomic regions identified by the LASSO method and by LLE or PCA methods we present Figures 10 and 11. The genomic region considered in Figure 10 is significantly enriched with both LLE-correlated and PCA-correlated SNPs, but shows no significance by LASSO. We observe substantial difference in genotype frequencies of each SNP within the region among the CEU, YRI and ASI samples in Figure 10. However, we also can observe in Figure 10 that in all three populations CEU, YRI and ASI there were large genotype frequency variation among SNPs in the region. On the whole region, we did not observe large differences in genotype frequency variation patterns among the CEU, YRI and ASI samples. Therefore, this region was assessed to be a structure un-informative region by the LASSO method. The genomic region on Chromosome 3 covering positions from 164,500,000 to 164,750,000 in Figure 11 is a structure informative region identified by the LASSO method, but not enriched with LLE-correlated or PCA-correlated SNPs. We do not observe large genotype frequency variation among SNPs in the CEU samples. In other words, the genotype frequencies at each SNP in the region were similar. However, we do observe significant genotype allele frequency variation in the YRI and ASI samples. This genotype frequency pattern was CEU population specific. This explains why this region was identified as a structure informative genomic region by the LASSO method, but not by the LLE or PCA method.

The human genomic variation is created by mutation and changed by biological, demographic and historical processes [27]. Genomic variation has been shaped by genetic drift and natural selection, and by the demographic history of our ancestors. To investigate the potential causes of the structure informative genomic regions and impact of natural selection on their formation, we scanned the CEU population for selection using Tajima D test. The number of most extreme 5% regions under selection is 1,080. We observe that 3.2%, 53.2% and 57.4% of the identified selection regions were overlapped with the structure informative genomic regions detected by the LASSO, LLE and PCA, respectively.

**Figure 12:**
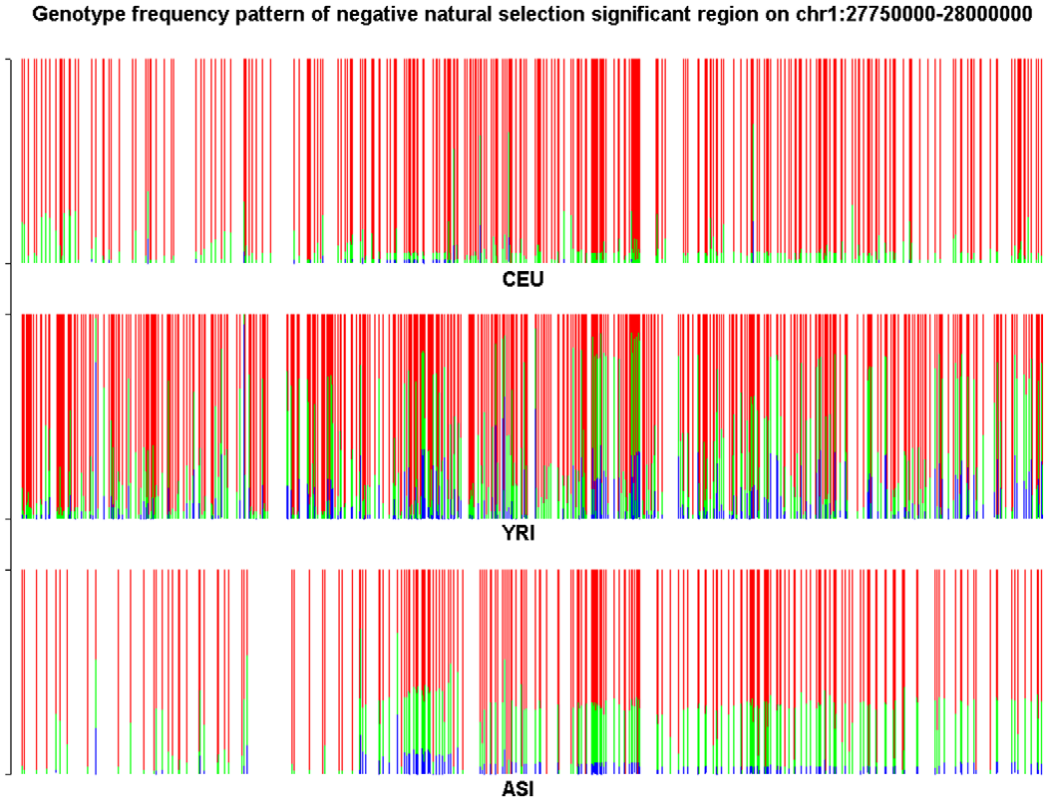
Genotype frequency pattern of the SNPs within the structure significantly informative genome region located on chromosome 1 between 27,750 kb and 28,000 kb under negative selection which was identified by all three methods: LASSO, LLE and PCA for CEU samples. The red color represents the homozygous genotype with the main allele, green color represents the heterozygous genotype and blue color represents the homozygous genotype with the minor allele.

**Figure 13:**
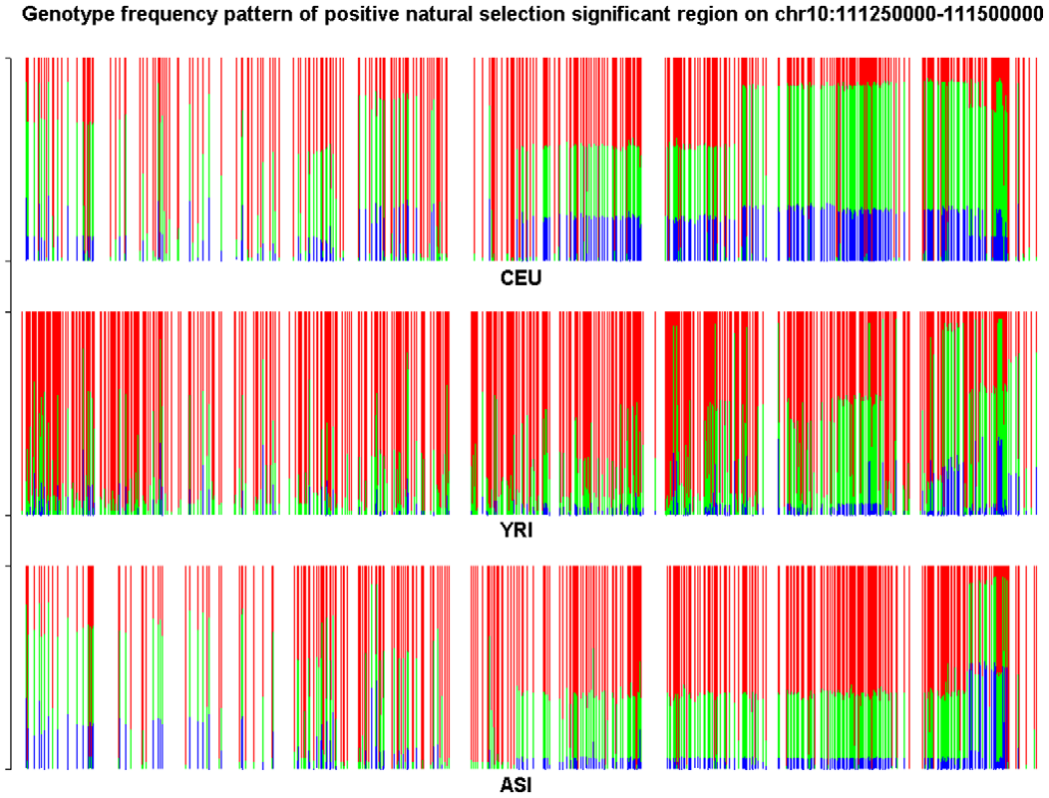
Genotype frequency pattern of the SNPs within the structure significantly informative genome region located on chromosome 10 between 111,250 kb and 111,500 kb under positive selection which was identified by all three methods: LASSO, LLE and PCA for CEU samples. The red color represents the homozygous genotype with the main allele, green color represents the heterozygous genotype and blue color represents the homozygous genotype with the minor allele.

To further illustrate that natural selection might be involved in the formation of structural informative genome regions, we present Figures 12 and 13 where the structure informative genomic regions (CEU specific) on Chromosomes 1 and 10 identified by all three methods: LASSO, LLE and PCA were overlapped with negative and positive selection regions in the CEU samples, respectively. Figure 12 shows that negative selection reduces variation in the region by eliminating some deleterious alleles in the CEU samples, while we observe in Figure 13 that positive selection caused local reduction in genetic diversity via increasing frequencies of favorable alleles in the population and “genetic hitchhiking”.

**Figure 14:**
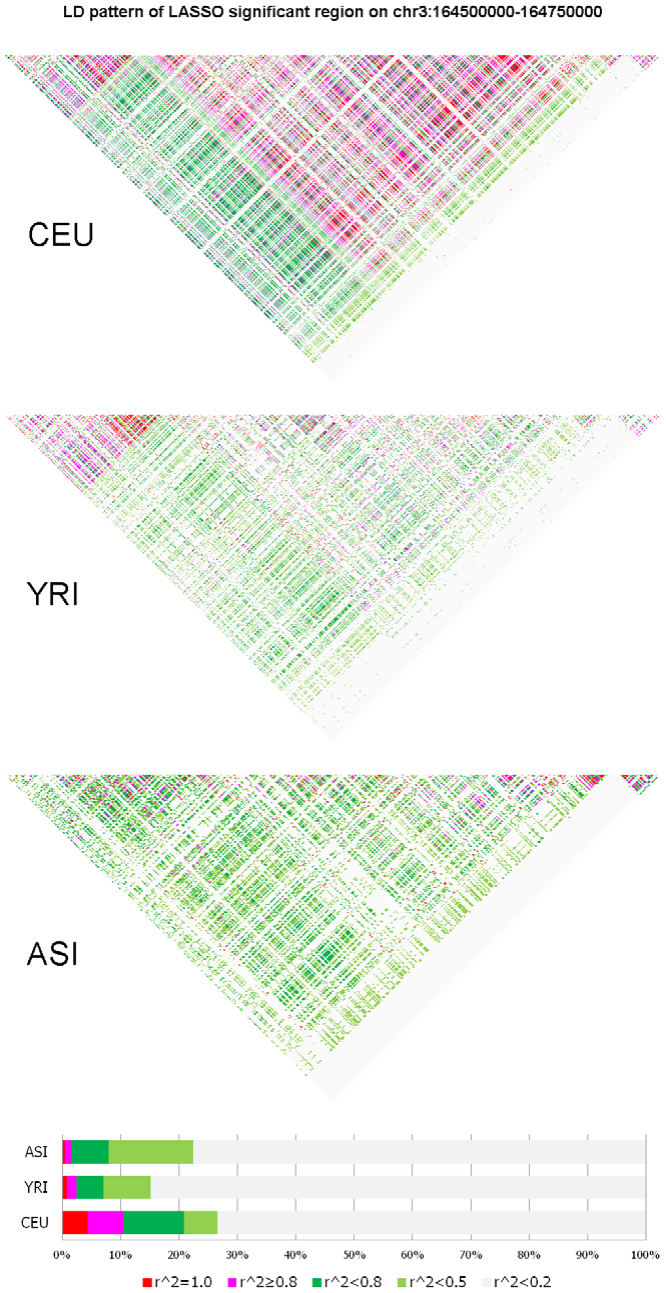
LD pattern in the structure significantly informative genome region for CEU samples, but not for YRI and ASI samples located on chromosome 3 between 164,500 kb and 164,750 kb which was identified by the LASSO method except the LLE or PCA method. The LD levels were measured by pair-wise 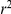
and illustrated by colors.

To further characterize the inherent features of the structural informative genomic regions, we study their LD patterns. The LD pattern is characterized by pair-wise 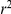 between SNPs. We consider the genomic region on Chromosome 3 between 164,500 kb and 164,750 kb which is identified to be a structure informative region for the CEU by the LASSO method. Figure 14 shows that a large proportion of SNPs in the CEU samples has a much stronger LD than that in the YRI and ASI samples. However, if the genomic region that is identified as structure uninformative, the LD levels across the region in all three populations CEU, YRI and ASI are very weak (Appendix S8). Since the LASSO regress the axis of population variation on the integral of genomic content within the region, it identifies collective association of the SNPs in the genomic region with the formation of structure. The many SNPs in the region as a whole undergo the same evolution events. These SNPs are co-evolved. The most SNPs in the structure informative region identified by the LASSO method will show a similar pattern. The existing LD among the SNPs in the region is kept in the evolution. The genotypes of SNPs in the structure informative region identified by the LASSO will be correlated and hence have a strong LD between them.

## Discussion

Emerging next-generation sequencing technologies which are evolving rapidly enable sequencing individual genomes and have the potential to discover the entire spectrum of sequence variations in a sample of individuals. The analysis of genome-wide sequencing data will provide new valuable tools for uncovering the evolution of populations, identifying the structure informative markers and genomic regions, improving our knowledge of population structure and developing personalized medicine. However, the development of efficient analysis tools for sequence-based population genetic studies is significantly lagging behind. Low frequency, high sequence errors, and missing data are three important challenges for the population genetic analysis of DNA sequence data. While often successful in uncovering population structure, PCA is sensitive to genotyping errors, outliers and missing values. As an alternative to PCA for population structure analysis, we used the LLE as a new low dimensional data representation tool to infer population structure and history. The population genetic analysis of next-generation sequencing data is currently in its infancy. The goal of this report is to stimulate discussion about the challenges for analyzing next-generation sequencing data in population structure and evolution studies.

We have shown several remarkable features of the LLE method for population structure and historical inference. First, the LLE was designed to discover a single global coordinate system in low dimensional space while preserving an important property that nearby data points in the high dimensional genomic space are co-projected to the low dimensional space. We showed that the reconstruction weights in the LLE which capture the topological properties of local neighborhoods of the subjects allow the estimation of relatedness structure among individuals and discover the pedigree tree of studied individuals. Second, we observed that in the presence of rare variants, the performance of the weighted genetic distance is better than Euclidean distance in the LLE analysis. Third, unlike PCA which is a linear reduction method, the LLE is a nonlinear dimension reduction algorithm. The LLE can efficiently extract information on an intrinsically low dimensional nonlinear graph embedding from high dimensional data space. The LLE has higher power to uncover population substructure. For instance in a study of 179 individuals from four populations YRI, CEU, CHB and JPT on 14,397,437 SNPs which were obtained from a low coverage pilot dataset in the 1000 Genomes Project, the individuals from the CHB and JPT can be completely separated by the LLE algorithms, but cannot be well separated by the PCA. Fourth, the LLE generates a sparse eigenvalue problem derived from a weighted graph which represents neighborhood relations, as opposed to the dense eigenvalue problems in PCA. The eigenvectors generated by both LLE and PCA can be used to define axes of genomic variation. However, the eigenvectors generated by the LLE can completely define the population axis. An interesting observation is that in a study of 179 individuals from a low coverage pilot dataset in the1000 Genomes Project, three eigenvectors with zero eigenvalue define the axes of populations: YRI, CEU and ASI. We can link each population to an eigenvector. Three eigenvectors can form three axes and an axis corresponds to a population. The PCA fails to have such representations. Fifth, the LLE can alleviate the impact of missing genotypes on the population structure inference. In the presence of rare variants, the true heterozygous are more likely to be called as missing. Biased estimation of genomic variation may introduce spurious population structure. Since the LLE algorithms only use pair-wise individual distance to construct neighborhood and genomic data of a few nearby individuals to calculate local reconstruction weights and low dimensional representation of the high dimensional genomic space, the impact of missing values and sequence errors on the LLE maps is limited. Therefore, the LLE is a better choice for sequence-based population structure inference than the PCA methods.

Identifying a set of markers that could be used for population structure and history inference will provide valuable information for population genetics and association studies. Similar to identifying PCA-correlated SNPs, we used a simple regression to search all LLE-correlated SNP markers that explicitly capture the structure of a population as identified by the LLE algorithms.

We used gene-set enrichment analysis to identify genomic regions which are enriched with either PCA-correlated SNPs or the LLE-correlated SNPs. Although traditional simple regression is successful for identifying PCA or LLE-correlated SNPs for structure inference one at a time, this method is mainly designed for common variants. While single SNP might make large contributions to the major axes of genomic variation individually, at the population level, the contribution of some individual SNP to the axes of genomic variation is too small to detect. The next-generation sequencing data are full of rare variants. Alternative to simple regions methods for identifying LLE or PCA-correlated SNPs and gene enrichment analysis for identifying genomic regions enriched with LLE or PCA-correlated SNPs, we have developed a new statistic that integrates genomic information content in a genomic region for collectively studying association of the genomic region with the low dimensional representation of the genomic data and used LASSO regression to search such regions across the genomes.

We have analyzed the entire set of SNPs including both common and rare variants in a low coverage pilot dataset in the 1000 Genomes Project. We observed that 25.1%, 44.9% and 21.4% of common variants were LLE-correlated SNPs and 31.8%, 44.2% and 22.3% of common SNPs were PCA-correlated, in the CEU, YRI and ASI populations, respectively. We found that the majority of rare variants ranging from 75.1% to 92.4% were LLE-correlated SNPs, which show that most rare variants are population specific. The number of ancestry informative SNPs identified by the LLE algorithms and PCA is close except for ASI where the number of ancestry informative rare SNPs identified by PCA is much smaller than that identified by the LLE algorithms. Interestingly, we observed the different allele frequency patterns between the top LLE-correlated and PCA-correlated SNPs. The top LLE-correlated SNPs contributing to the axis of genetic variation in the CEU were CEU specific. In other words, samples from the CEU showed significant DNA variation at the top LLE-correlated SNPs. However, samples from the YRI and ASI did not show any DNA variation at these top-LLE correlated SNPs. However, both top PCA-correlated common and rare SNPs did not show such population specific features. We observed that these SNPs showed DNA variations at least in two populations.

We observed that the number of the LLE-correlated SNP or PCA-correlated SNP enriched genomic regions is almost sixteen times than that identified by the LASSO method. We observed substantial differences in allele frequency and LD patterns between the LLE-correlated SNP or PCA-correlated SNP enriched genomic regions, and LASSO identified structure informative genomic regions. In the LLE-correlated or PCA-correlated SNP enriched genomic regions, we observed not only large variation in genotype frequency at single SNPs in the region among three populations, but also large genotype frequency variation among SNPs across the genome in all three populations. However, in the LASSO identified structure informative genomic regions that are specific for the CEU, the genotype frequencies at almost all SNP in the region were similar in the CEU samples, but genotype allele frequency variation among SNPs in the YRI and ASI samples were significant. It is also observed that a large proportion of the structure informative genomic regions identified by the LLE, PCA and LASSO methods are not overlapped. This shows that these three methods are complimentary.

Interestingly, we found that a large proportion of identified selection regions ranging from 3.2% to 57.4% were overlapped with the structure informative genomic regions. This implies that natural selection might contribute to the formation of population structure. We also observed that both negative and positive natural selection may lead to local reduction in genetic diversity and cause differences in the genotype frequency and LD patterns between populations.

Although our results are preliminary due to limitations of available next-generation sequence data, the concepts and methods described in this report are expected to emerge as an alternative analytic framework to current methods for population structure and historical inference and should stimulate further discussions in addressing the great challenges raised by next-generation sequencing technologies for population genetic studies.

A program for implementing the developed statistics can be downloaded from our website http://www.sph.uth.tmc.edu/hgc/faculty/xiong/index.htm.

## Materials and Methods

### Problem Formulation for the Low Dimensional Representation of High Dimensional Genomic Data by the LLE

The dimension in the genomic variant data space is very high. To alleviate the curse of dimensionality, it is indispensable to transform the data from the original high dimensional space to a low dimensional space. The LLE algorithm is a recently developed algorithm [28]. It is an extension of mixture models for local dimensionality reduction which perform PCA within each cluster [29]. The LLE algorithm attempts to project a nonlinear manifold in a high dimensional space onto the desired low dimensional representation with the property that nearby data points in the high dimensional space remain nearby in the low dimensional space.

We assume that *n* individuals are sampled. We consider *p* SNPs. In general, *p* is much larger than *n*. Values of 0, 1, and 2 are assigned to the indicator variable *x_ij_* for the genotypes AA, Aa and aa at the j-th SNP of the i-th individual, respectively. Let 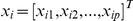 be a vector of the indicator variables for the i-th individual which represent the genetic information for the i-th individual. We define the data matrix 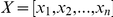 with dimension *n* 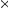 *p*. The data *X* can be represented by an undirected weighted graph *G* = {*X,W*}, where each node denotes an individual and weight associated with an edge measures the similarity between a pair of nodes (a pair of individuals). Given a set of genotype data 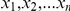 in *R^p^*, find a set of points 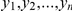 in the low dimensional space *R^m^* (*m*<<*p*) which best preserves the similarity relationship between the individuals in the original high dimensional data space. To achieve this goal, the LLE algorithm consists of three steps. The first step is to search the nearest neighbors of each data point. The second step is to compute the weights that best reconstruct each data point from its neighbors. The third step is to find the desired low-dimensional coordinates in a non-linear dimensional projection 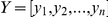 of the original high dimensional data 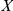. Next we introduce three steps in detail.

### Neighborhood Construction

Before searching nearest neighbors, we should develop a statistic to measure the distance between two data points. The widely used geometric distance between two data points is the Euclidean distance. Euclidean distance is similar to the genetic distance between individuals defined as the number of alleles shared by state (IBS) [30]. Other genetic distance measures between individuals can also be used to quantify the genetic similarity between data points (individuals) [31], [32]. To adjust for impact of allele frequency on the genetic distance we can incorporate allele-specific weights into the genetic distance measure. Let 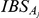 and 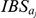 be the number of copies of alleles 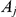 and 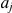 at the *j*-th SNP shared by the pair of individuals, respectively. Then, the allele frequency weighted genetic distance can be defined as
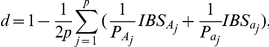
where 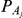 and 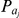 are the frequencies of the alleles 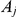 and 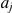, respectively.

Now we are in a position to introduce the neighbors of each data point [29], [33]. The simplest formulation of the algorithm is to find a fixed number of nearest neighbors per data point. Let *k*<<*p* be the number of data points in a neighborhood and 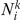 denote the neighborhood of the data point 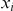 in the high dimensional data space that includes only its *k* nearest data points, as measured by genetic distance (each data point may chose a different size *k* of its neighborhood). In the graphical representation of the data, nodes *i* and *j* are connected by an edge, if *i* is among *k* nearest neighbors of *j* or *j* is among *k* nearest neighbors of *i*. The size *k* of the neighborhood has a large impact on the performance of the LLE algorithm. The size *k* should be small enough to make the approximate manifold locally linear, but also must be sufficiently large to recover embedding. An implementation of LLE should ensure that the graph formed by linking each data point to its neighbors is connected. Otherwise, the LLE algorithm should be applied separately to each connected component.

### Local Reconstruction Weight Calculation

The second step of the LLE algorithm is to reconstruct each data point by a linear combination of its nearest *k* neighbors:
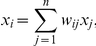
where 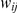 is a scalar weight, 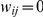 if 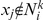 and 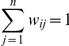. Let 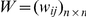 be a sparse weight matrix. We define the reconstruction error as
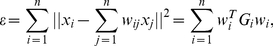
(1)where 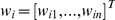 and 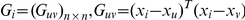, is a symmetric, nonnegative definite matrix. The optimal reconstructed weights can be obtained by minimizing the reconstruction error under the constraint: 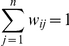. The optimal solution is given by
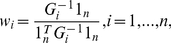
(2)where 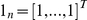
and it is understood that for 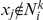 the corresponding weight 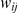 is zero. The solution (2) requires an explicit inverse of the matrix 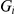. In practice, to avoid inverse computing, the same results can be obtained by solving the linear system of equations:
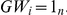
The final optimal weights can be obtained by rescaling the weights to sum to one.

### Eigenvalue Problem

The final step of the LLE algorithm is to compute the low dimensional representation *Y* while preserving the neighborhood relationship among the data points in the original high dimensional data space. Since the local construction weights measure the neighborhood relations among the data points in the original high dimensional data space, the low dimensional representation *Y* can be identified by minimizing the following graph embedding cost function:
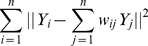
(3)subject to the constraints: 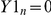 and 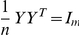. The cost function in equation (3) can be obtained by replacing 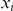 by 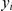 in the equation (1). Instead of optimizing the weights in equation (1), we optimize the low dimensional representation 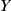 in the graph embedding the cost function while keeping the weights found in equation (1) fixed. In other words, we attempt to find low dimensional representation 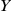
that are reconstructed by the same weights as that for the high dimensional data 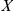. Let 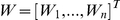, 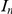 be an identity matrix and 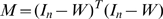. Then equation (3) can be reduced to
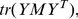
(4)where tr denotes a trace of the matrix. Therefore, the low dimensional representation 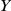 can be identified by solving the constrained minimization problem:
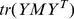
(5)s.t. 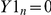 and 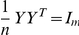.

There are many ways to solve this optimization problem. One way that has a close connection to PCA and is also most straightforward is to find the bottom 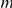 eigenvectors of the matrix M as the desired low dimensional representation 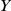 (see Appendix S9).

Why would we expect that the LLE will preserve the neighborhood structure of the graph embedding in the low dimensional space? For simplicity, we explain the one dimensional case. We assume that 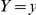 is a vector of low dimensional representation. Then, we have
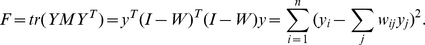
If 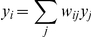 which implies that each data point in the low dimensional space can be restructured from the same neighbors with the same weights as that in the original dataset then the objective function 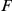 is minimized over all vectors. The neighborhood relationships among the data points reflect the population structure. If vectors that minimize the objective function 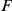 are chosen then they will allow revealing of the population structure.

The LLE for the population genetic studies has several remarkable features (Appendix S2 and Appendix S10 [34]). First, we can show that both weights and low dimensional coordinates of nonlinear dimensional project are a function of kinship coefficients or the probability of individual sharing IBD. Therefore, the LLE algorithms will provide a useful tool for identifying the genetic relationships among the individuals and population structure.

Second, if the number of 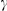
+1 eigenvalues of the matrix 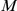
is equal to zero then the number of subpopulations in the studies is 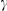. Third, the eigenvalues measure the reconstruction errors. We can show that the eigenvalue is the average square of the locally linear reconstruction error in the low dimensional embedding space. The eigenvalue zero indicates that the each low dimensional coordinates can be completely, linearly reconstructed by its neighborhood data points. Fourth, the degree of population substructure can be measured by the trace of between-class covariance matrix. If 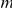 subpopulations are completely separated, then their separability is defined as
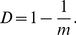
(6)If subpopulations cannot be completely separated, the measure of separability of subpopulations is defined as
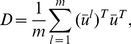
(7)where 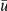 is a vector of the average of 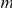 low dimensional representation of the original genetic data in the high dimensional data space of 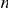 individuals.

The LLE and PCA have close relationships. PCA seeks projection direction with maximal variance or to remove the projection directions with minimal variance [14]. PCA can be formulated (Appendix S11) as
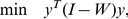
(8)where 
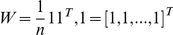
, 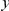
is a vector. Equation (8) implies that under the graph embedding framework for dimension reduction of the graph embedding underlying PCA has the intrinsic graph structure with all nodes connected and equal weights associated with the edges. If we define the matrix 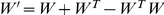 and consider a row vector 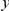
of the data matrix 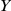, the equation (5) is reduced to
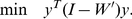
(9)Equation (9) has the similar form as that in equation (8), but with the complex weight matrix 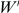 which has neighborhood structure. Unlike PCA whose graph embedding connects all the nodes in the graph and hence does not have neighborhood structure, the LLE projects high dimensional data to a desired low dimensional space while optimally preserving local neighborhood information. However, we should also point out that PCA gives a very natural ranking, whereas LLE-like methods may not so.

### Identification of Ancestry-Informative Markers and Genomic Regions

The low-dimensional coordinates in a non-linear dimensional projection of genomic variants of an individual are summary statistics. Although they unravel the patterns of genomic variation in populations and uncover the population substructure the low dimensional representation of genomic data do not provide information about what genomic variations and regions influence the formation of subpopulations. It is useful to identify SNPs or genomic regions associated with the low dimensional representation of the high dimensional genomic data which can provide information on local changes in ancestry across the genome. Emerging next-generation sequencing technologies enable sequencing individual genomes and have the potential to discover the entire spectrum of sequence variations in a sample of individuals including common and rare variants [1], [35]–[41]. A popular method for identifying ancestry-informative markers in the PCA is a simple regression where the principal component score is regressed on a genomic variant variable. Although an individual rare variant may have a large genetic effect, at the population level, their genetic effect is too small to detect. In addition, new sequence technologies are highly error prone. To overcome these limitations, we propose to integrate genomic information content in a genomic region for collectively testing association of the genomic region with the low dimensional representation of the genomic data. Let 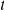 be the position of a genetic variant along a chromosome or within a genomic region and 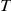 be the length of the genomic region being considered. For convenience, we rescale the region 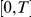 to 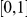; because the density of genetic variants is high, we can view 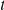 as continuous. We assume that the whole genome is grouped into 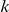 genomic regions 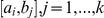. We define the indicator variable for the genotype of the i-th individual at the genomic location 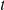 in the genomic region 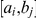 as
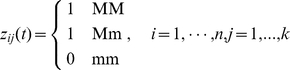
(10)where M is a minor allele at the genomic position 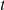. The genotypic profiles 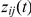 contain the information of the genetic variation and its relative genomic position. For convenience of discussion, 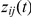 are referred to as the information content at the genomic position *t*. Their integral 
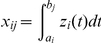
 summarizes the information content of the genome in the region 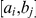. It is a useful measure of the genetic variability of a genome region. We assume that the variable 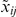 are standardized:
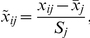
where 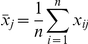 and 
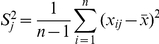
. Thus, we have
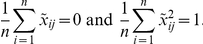
The LASSO regression that uses a 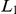 penalty to achieve a sparse solution will be used to identify ancestry-informative genomic regions which characterize local changes in ancestry. The LASSO is to solve the following problem:
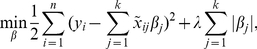
(11)where 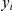
is the low dimensional coordinate of the genomic data for the *i*-th individual in the nonlinear dimensional project, 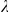 is a penalty parameter, and 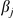 are regression coefficients. A solution by a successive coordinate-wise descent algorithm for optimization problem (11) is given by [42]

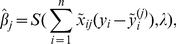
(12)where 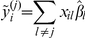, 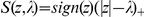, 
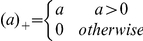
. The genomic regions with 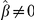 are considered ancestry-informative regions which provide information on local changes in ancestry across the genome. After the ancestry-informative genomic regions are identified, a simple regression will be used to test association of each SNP in the ancestry-informative region.

## Supporting Information

Appendix S1 **Three eigen-vectors in the eigen-space of zero eigen-value for the nonlinear dimensional mapping of all 14,397,437 SNPs of 179 individuals from four populations YRI, CEU, CHB and JPT mapped by the LLE using the Euclidean distance.** The corresponding coordinate of the eigenvector associated with individuals from YRI, CEU, and CHB and JPT (ASI) was mapped to the x axis, the y axis and z axis, respectively.
(TIF)

Appendix S2 **Proof A.**
(DOC)

Appendix S3 **The differences between the weights estimated by the original genomic data, and the low dimensional coordinates and the difference between the weights by the original genomic data and the PCA as a function of the weights arranged by their location in the weight matrix for 60 individuals from CEU population.**
(TIF)

Appendix S4 **DNA variation pattern of the LLE-correlated, YRI specific SNPs across populations.**
(DOC)

Appendix S5 **DNA variation pattern of the LLE-correlated, ASI specific SNPs across populations.**
(DOC)

Appendix S6 **The distribution of structural informative genomic regions identified by the LASSO, LLE and PCA for YRI samples.** The genome was divided into nonoverlapping 250 kb bins (*x* axis), and the *y* axis represents the P-value for testing whether the genomic region is significantly structure informative.
(TIF)

Appendix S7 **The distribution of structural informative genomic regions identified by the LASSO, LLE and PCA for ASI samples.** The genome was divided into nonoverlapping 250 kb bins (*x* axis), and the *y* axis represents the P-value for testing whether the genomic region is significantly structure informative.
(TIF)

Appendix S8
**LD pattern in the structure significantly informative genome region for CEU samples, but not for YRI and ASI samples located on chromosome 13 between 66,000 kb and 66,250 kb which was identified by the LLE and PCA method except the LASSO method.** The LD levels were measured by pair-wise 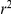
and illustrated by colors.(TIF)

Appendix S9 **Proof B.**
(DOC)

Appendix S10 **Proof C.**
(DOC)

Appendix S11 **Proof D.**
(DOC)
