# Supplementary figures and images for: Manifold Learning for Human Population Structure Studies
Source: PLoS One. 2012 Jan 17;7(1):e29901. doi: 10.1371/journal.pone.0029901 (PMC3260176; doi:10.1371/journal.pone.0029901)

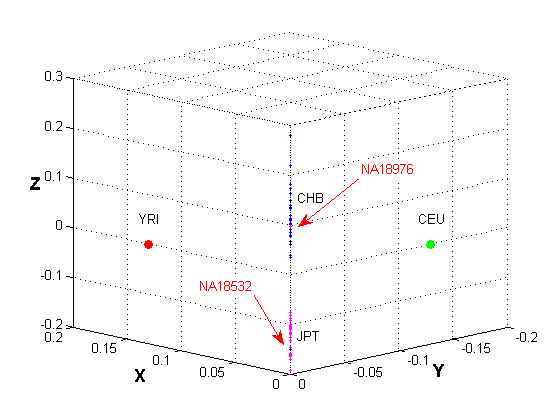

Supplement: Appendix S1 — Three eigen-vectors in the eigen-space of zero eigen-value for the nonlinear dimensional mapping of all 14,397,437 SNPs of 179 individuals from four populations YRI, CEU, CHB and JPT mapped by the LLE using the Euclidean distance. The corresponding coordinate of the eigenvector associated with individuals from YRI, CEU, and CHB and JPT (ASI) was mapped to the x axis, the y axis and z axis, respectively. (TIF) [file pone.0029901.s001.tif]

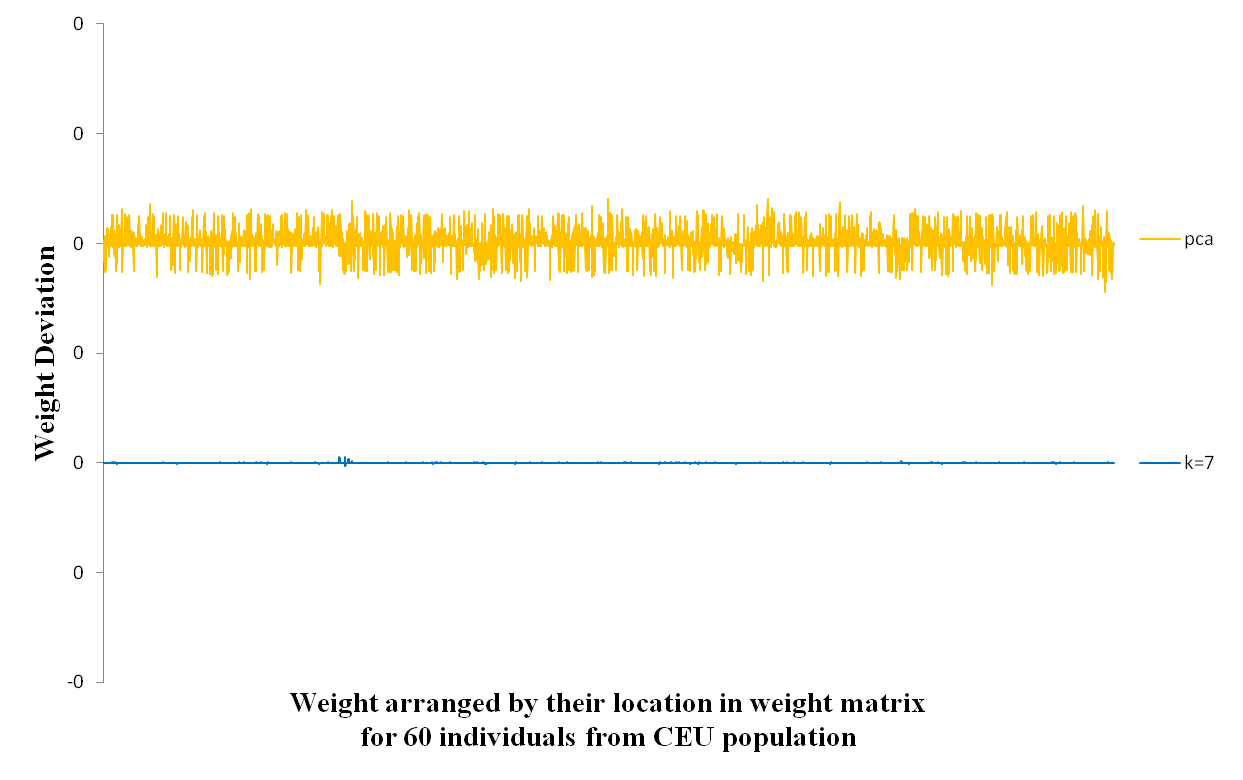

Supplement: Appendix S3 — The differences between the weights estimated by the original genomic data, and the low dimensional coordinates and the difference between the weights by the original genomic data and the PCA as a function of the weights arranged by their location in the weight matrix for 60 individuals from CEU population. (TIF) [file pone.0029901.s003.tif]

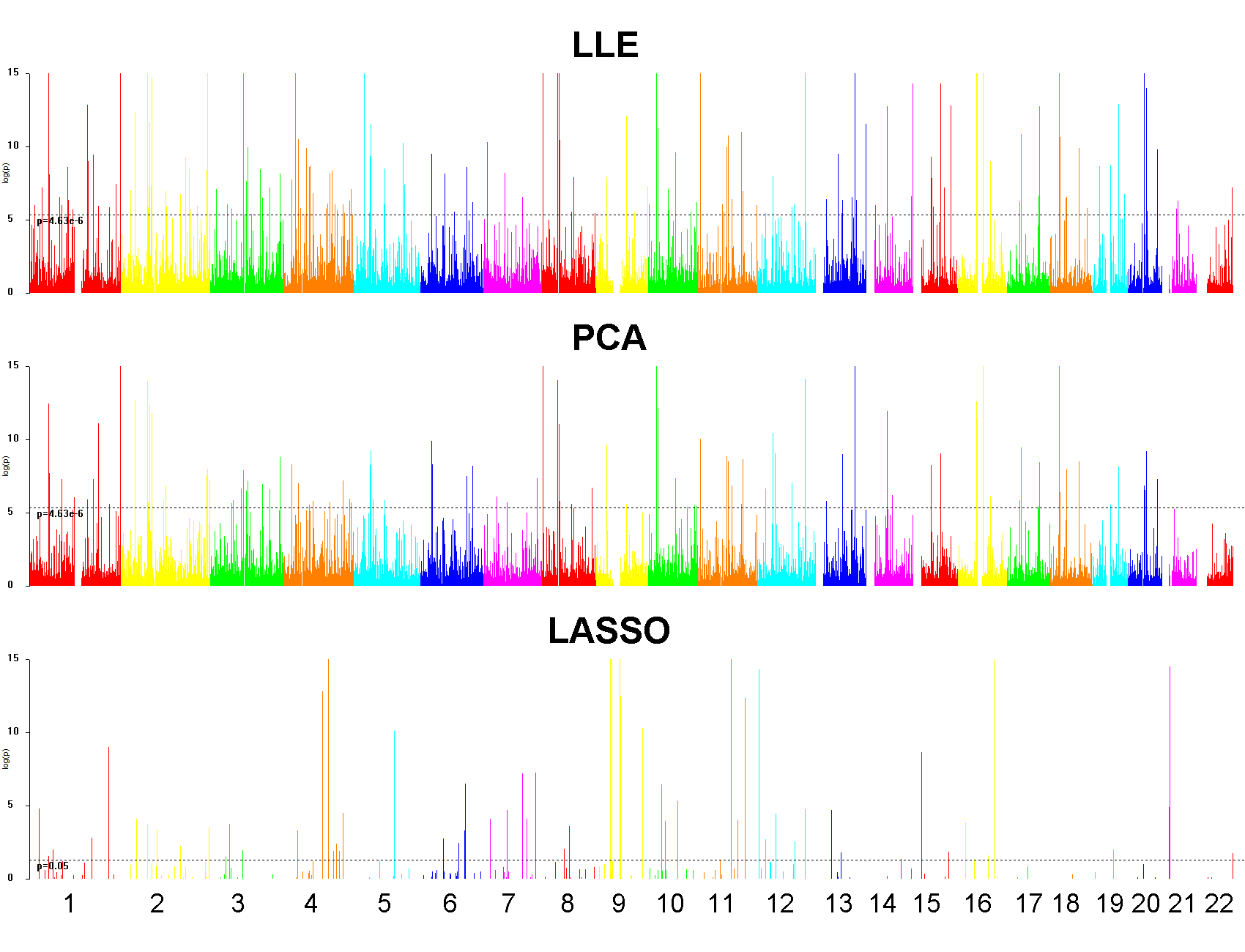

Supplement: Appendix S6 — The distribution of structural informative genomic regions identified by the LASSO, LLE and PCA for YRI samples. The genome was divided into nonoverlapping 250 kb bins (x axis), and the y axis represents the P-value for testing whether the genomic region is significantly structure informative. (TIF) [file pone.0029901.s006.tif]

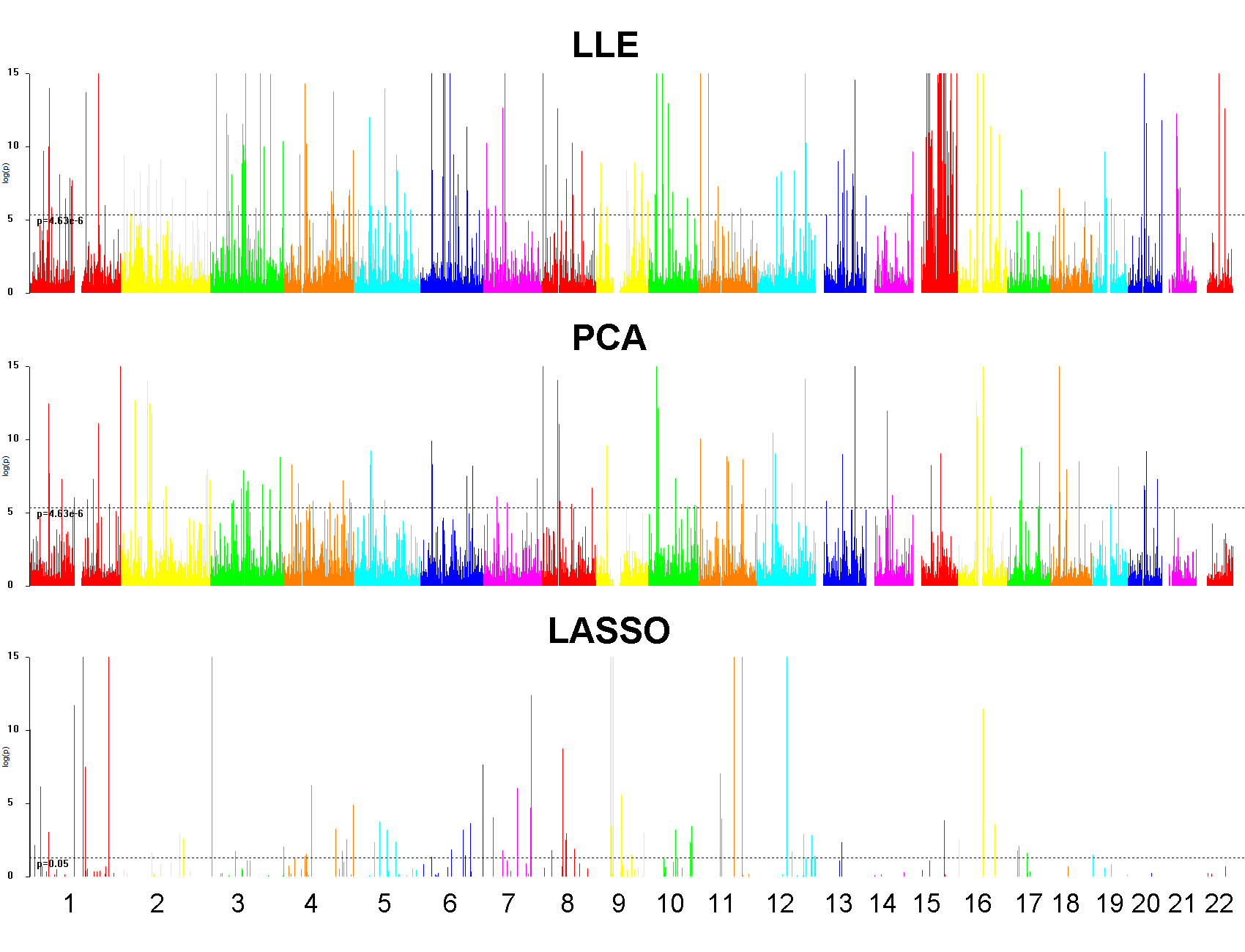

Supplement: Appendix S7 — The distribution of structural informative genomic regions identified by the LASSO, LLE and PCA for ASI samples. The genome was divided into nonoverlapping 250 kb bins (x axis), and the y axis represents the P-value for testing whether the genomic region is significantly structure informative. (TIF) [file pone.0029901.s007.tif]

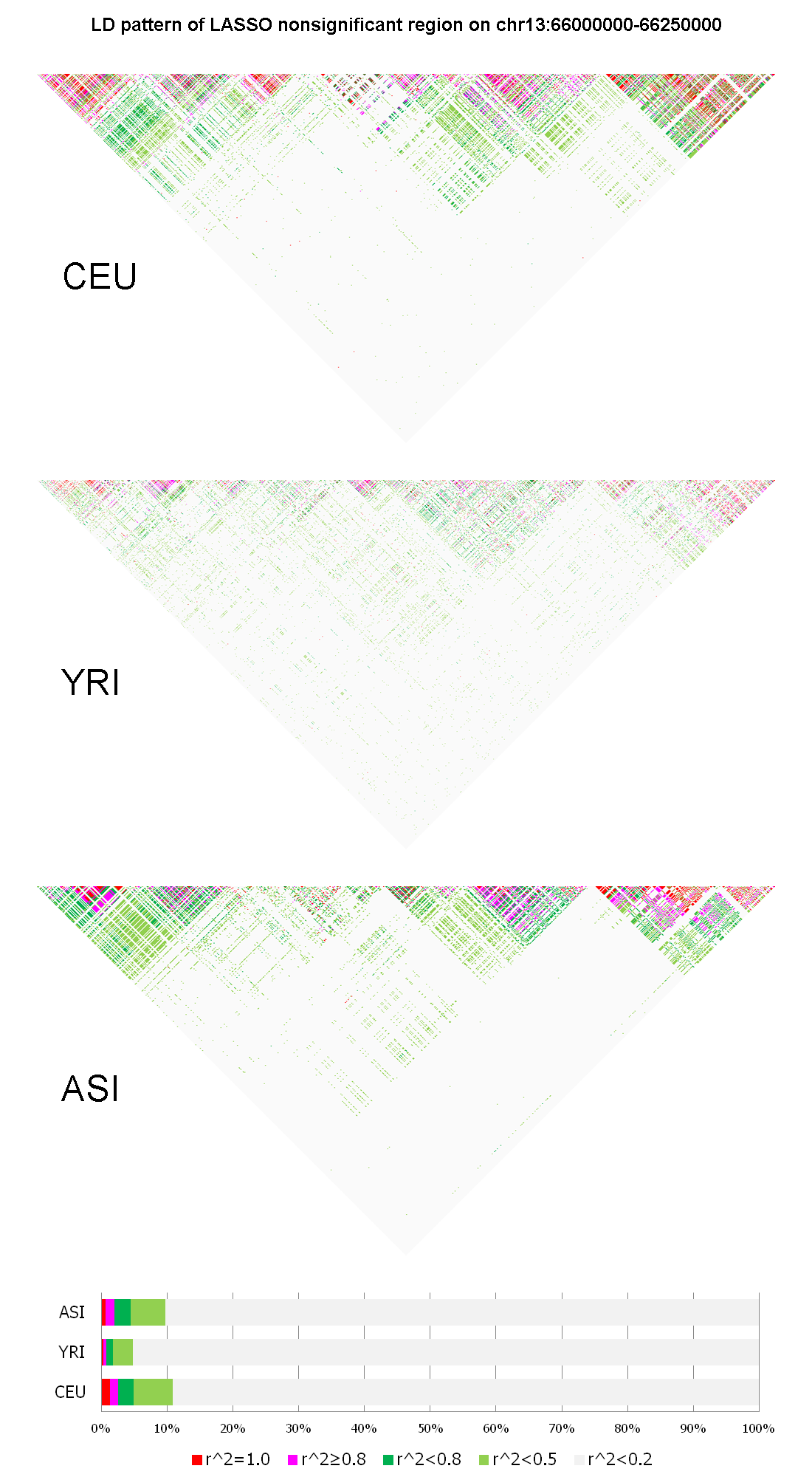

Supplement: Appendix S8 — LD pattern in the structure significantly informative genome region for CEU samples, but not for YRI and ASI samples located on chromosome 13 between 66,000 kb and 66,250 kb which was identified by the LLE and PCA method except the LASSO method. The LD levels were measured by pair-wise and illustrated by colors. (TIF) [file pone.0029901.s008.tif]
