# Supplementary material for: Manifold Learning for Human Population Structure Studies
Source: PLoS One. 2012 Jan 17;7(1):e29901. doi: 10.1371/journal.pone.0029901 (PMC3260176; doi:10.1371/journal.pone.0029901)
Supplement: Appendix S2 — Proof A. (DOC) [file pone.0029901.s002.doc]

**Proof A**

First we show that the weights are a function of kinship coefficients, for the simplicity of discussion, let *x* be a data point that can be represented as a linear combination of its neighborhood points:

$x=\sum_{j=1}^{k} w_{j}x_{j}$ (A1)

where *k* is the number of its neighbors, *w_j_* is the weight, *x_j_* is the j-th neighborhood. Taking inner product with *x_i_* on both sides of equations, we obtain:

$\sum_{j=1}^{k} \left\langle x_{j},x_{i}\right\rangle w_{j}=\left\langle x,x_{i}\right\rangle$ (A2)

where < *x_j_* , *x_i_* > is the inner product between the data points *x_j_* and *x_i_*. If *x* is coded as

$x=\left\{\begin{matrix} 1 & A \\ 0 & a \end{matrix}\right.$ (A3)

where *A* and a are two alleles at the data point *x*.

Let *p* be the frequency of allele *A*, then we obtain Ex = p, Ex^2^ = p, and Var(x) = p(1-p). If *x* is coded as

$x=\left\{\begin{matrix} \begin{matrix} 0 & aa \end{matrix} \\ \begin{matrix} 1 & Aa \end{matrix} \\ \begin{matrix} 2 & AA \end{matrix} \end{matrix}\right.$ (A4)

then, we have Ex = 2p and Var(x) = 2p(1-p). The indicator variable for coding the genotypes in equation (A4) is normalized as:

$x=\left\{\begin{matrix} \begin{matrix} \frac{-2p}{\sqrt{2p(1-p)}} & aa \end{matrix} \\ \begin{matrix} \frac{1-2p}{\sqrt{2p(1-p)}} & Aa \end{matrix} \\ \begin{matrix} \frac{2(1-p)}{\sqrt{2p(1-p)}} & AA \end{matrix} \end{matrix}\right.$ (A5)

then, Ez=0 and Var(z) = 1. The inner product <*z_j_*,*z_i_*> is then expressed as

$\left\langle z_{j},z_{i}\right\rangle=\frac{1}{L}\sum_{l=1}^{L} \frac{(x_{je}-2p_{e})(x_{ie}-2p_{e})}{2p_{e}(1-p_{e})}\approx2\varphi_{ij}$ (A6)

where *ф_ij_* is the kinship coeffient between the i-th individual and j-th individual.

Equation (A2) can be rewritten as

$\left[\begin{matrix} 1+h_{1} & 2\varphi_{12} & \cdots& 2\varphi_{1k} \\ 2\varphi_{21} & 1+h_{2} & \cdots& 2\varphi_{2k} \\ \vdots& \vdots& \ddots& \vdots\\ 2\varphi_{k1} & 2\varphi_{k2} & \cdots& 1+h_{k} \end{matrix}\right].\left[\begin{matrix} w_{1} \\ w_{2} \\ \vdots\\ w_{k} \end{matrix}\right]=\left[\begin{matrix} 2\varphi_{x1} \\ 2\varphi_{x2} \\ \vdots\\ 2\varphi_{xk} \end{matrix}\right]$ (A7)

which implies that the weights are a function of kinship coefficients.

Suppose that the sampled individuals come from independent nuclear families with unrelated father and mother and one child. Then, the indicator variable for the child can be written as

$$x_{c}=w_{1}x_{f}+w_{2}x_{m}$$

then, we have

$w_{cf}=\frac{2\varphi_{fc}}{1+h_{f}}$ and $w_{cm}=\frac{2\varphi_{mc}}{1+h_{m}}$ (A8)

where *ф_fc_* and *ф_mc_* are kinship coefficients between father and his child, between mother and her child, respectively.

Similarly, we can obtain

$w_{fc}=\frac{2(1+h_{m})\varphi_{fc}}{(1+h_{c})(1+h_{m})-4\varphi_{mc}^{2}}$,$w_{fm}=\frac{-4\varphi_{fc}\varphi_{mc}}{(1+h_{c})(1+h_{m})-4\varphi_{mc}^{2}}$

$w_{mc}=\frac{2(1+h_{f})\varphi_{mc}}{(1+h_{f})(1+h_{c})-4\varphi_{fc}^{2}}$ and $w_{mf}=\frac{-4\varphi_{fc}\varphi_{mc}}{(1+h_{f})(1+h_{c})-4\varphi_{mc}^{2}}$

The matrix *M* for seeking the low dimensional coordinates is of the following form

$$M=\left[\begin{matrix} M_{1} & 0 & \cdots& 0 \\ 0 & M_{2} & \cdots& 0 \\ \vdots& \vdots& \ddots& \vdots\\ 0 & 0 & \cdots& M_{n} \end{matrix}\right]$$

Where n is the number of nuclear families. The matrix *M_j_* is given by

$$M_{j}=\left[\begin{matrix} 1+w_{mf}^{2}+w_{cf}^{2} & -w_{fm}-w_{mf}+w_{fm}w_{cf} & -w_{fc}-w_{cf}+w_{mf}w_{mc} \\ -w_{fm}-w_{mf}+w_{fm}w_{cf} & 1+w_{fm}^{2}+w_{cm}^{2} & -w_{mc}-w_{cm}+w_{fm}w_{fc} \\ -w_{fc}-w_{cf}+w_{mc}w_{mf} & -w_{mc}-w_{cm}+w_{fc}w_{fm} & 1+w_{fc}^{2}+w_{mc}^{2} \end{matrix}\right]$$

where *w_fm_*, *w_mf_*, *w_fc_*, *w_cf_*, *w_mc_* and *w_cm_* are given for the j-th family.

The components of the eigenvectors (or low dimensional coordinates) corresponding to the j-th family are all equal to zero except for corresponding to the individuals in the j-th family where the components are the function of the kinship coefficients between the individuals in the j-th family. Therefore, the low dimensional coordinates are determined relationships among the individuals. This conclusion will hold for the more complicated pedigree structures.
