# Supplementary material for: Manifold Learning for Human Population Structure Studies
Source: PLoS One. 2012 Jan 17;7(1):e29901. doi: 10.1371/journal.pone.0029901 (PMC3260176; doi:10.1371/journal.pone.0029901)
Supplement: Appendix S4 — DNA variation pattern of the LLE-correlated, YRI specific SNPs across populations. (DOC) [file pone.0029901.s004.doc]

**Supplementary Table S1.** DNA variation pattern of the LLE-correlated, YRI specific SNPs across populations.

Raw with star denotes rare alleles (MAF ≤5%), raw without star denotes common alleles (MAF>5%); (LLE) minimum or (PCA) minimum means SNPs choosing by the smallest p-value; (LLE) bonferroni or (PCA) bonferroni means SNPs choosing by the p-value around bonferroni test; (LLE) maximum or (PCA) maximum means SNPs choosing by the highest p-value.
