# Supplementary material for: Manifold Learning for Human Population Structure Studies
Source: PLoS One. 2012 Jan 17;7(1):e29901. doi: 10.1371/journal.pone.0029901 (PMC3260176; doi:10.1371/journal.pone.0029901)
Supplement: Appendix S9 — Proof B. (DOC) [file pone.0029901.s009.doc]

**Appendix B**

Let

$Y=[y_{1},...,y_{n}]=[u_{1},...,u_{m}]^{T.}$.

Then, we have

$tr(YMY^{T})=\sum_{i=1}^{m} u_{i}^{T}Mu_{i}$.

The optimization problem (5) can be reduced to

Min $F=\sum_{i=1}^{m} u_{i}^{T}Mu_{i}$ (B1)

s.t. $u_{i}^{T}1_{n}=0,\frac{1}{n}u_{i}^{T}u_{i}=1,u_{i}^{T}u_{k}=0,i=1,...,m,i<k,k=2,...m$.

For each $i$, we seek $u_{i}$ to minimize

$F_{i}=u_{i}^{T}Mu_{i}$ (B2)

s.t. $\frac{1}{n}u_{i}^{T}u_{i}=1,u_{i}^{T}1_{n}=0,u_{i}^{T}u_{k}=0,i<k.$

Using the Lagrangian multiplier to solve optimization problem (B2), we obtain

Min $u_{i}^{T}Mu_{i}+\lambda(n-u_{i}^{T}u_{i})-\mu u_{i}^{T}1_{n}$ (B3)

Therefore, the solution for optimization of the problem must satisfy

$Mu_{i}=\lambda u_{i}+\mu1_{n}$. (B4)

If we assume $\lambda=0$and $\mu=0$then since $M1_{n}=0$, the unit vector $1_{n}=[1,...,1]^{T}$satisfies equation (B4) and hence is a optimal solution to problem (B3). Consider the following eigen equation:

$Mu_{i}=\lambda u_{i}$. (B5)

The unit vector $1_{n}$is also an eigenvector corresponding eigenvalue of zero in eigenequation (5). Since the remaining eigenvectors are orthogonal to the unit vector, the solutions to eigenequation (B5) are the solutions to optimization problems (B2) and (B1). If we assume the dimension of low dimensional space is $m$ we then select the smallest $m$eigenvectors of the matrix $M$as our desired low dimensional representation $Y$of the original high dimensional data$X$.
