# Supplementary material for: Manifold Learning for Human Population Structure Studies
Source: PLoS One. 2012 Jan 17;7(1):e29901. doi: 10.1371/journal.pone.0029901 (PMC3260176; doi:10.1371/journal.pone.0029901)
Supplement: Appendix S10 — Proof C. (DOC) [file pone.0029901.s010.doc]

**Proof C**

Assume there are $\gamma$ subpopulations. By exchanging the rows and columns of the weight matrix $W$ we can obtain its block-diagonal structure:

$W=\left[\begin{matrix} W_{1} & 0 & ... & 0 \\ 0 & W_{2} & ... & 0 \\ ... & ... & ... & ... \\ 0 & 0 & ... & W_{\gamma} \end{matrix}\right]$,

which implies that the $M$is a $\gamma$block-diagonal matrix:

$M=\left[\begin{matrix} (I_{1}-W_{1})^{T}(I_{1}-W_{1}) & 0 & ... & 0 \\ 0 & (I_{2}-W_{2})^{T}(I_{2}-W_{2}) & ... & 0 \\ ... & ... & ... & ... \\ 0 & 0 & ... & (I_{\gamma}-W_{\gamma})^{T}(I_{\gamma}-W_{\gamma}) \end{matrix}\right]$.

Assume that the dimension of the matrix $W_{i}$is $n_{i}$. Let $1_{n_{i}}=[1,...,1]^{T}$be a unit vector with $n_{i}$components. Recall that summation of each row in the matrix $W_{i}$is equal to 1, which implies that $(I_{i}-W_{i})1_{n_{i}}=1_{n_{i}}-W_{i}1_{n_{i}}=1_{n_{i}}-1_{n_{i}}=0$. Let $0_{n_{j}}=[0,...,0]^{T}$be a vector of zero with $n_{j}$components. Define a vector $u_{i}=[0_{n_{1}},...,1_{n_{i}},...,0_{n_{\lambda}}]^{T},i=1,...,\gamma$, Then, we have

$Mu_{i}=[0_{n_{1}},...,0_{n_{i}},...,0_{n_{\gamma}}]^{T}=0u_{i}$, which implies that the vector $u_{i}$is an eigenvector corresponding to the eigenvalue of zero. Also, we note that $M1_{n}=0_{n}$. Therefore, $1_{n}$is also an eigenvector corresponding to eigenvalue of zero. Thus, the total number of eigenvectors corresponding to eigenvalue of zero is $\gamma+1$.

Multiplying equation (A5) on both sides by $u_{i}$, we obtain

$\lambda=\frac{1}{n}u_{i}^{T}Mu_{i}$, (C1)

which implies that eigenvalue is the average square of the locally linear reconstruction error in the low dimensional embedding space. The eigenvalue zero indicates that the each low dimensional representation can be completely, linearly reconstructed by its neighborhood data points.

The between population separability can be measured by trace of between-class variance matrix. If $m$ subpopulations are completely separated, then their separability is given by

, (C2)

where $\bar{u}_{l}=[0,..,1,0,..0]^{T}$and $\bar{u}=\frac{1}{m}[1,1,...,1]^{T}$.

If subpopulations cannot be completely separated, then we define

,

where $m$is the dimensionality of the low dimensional space and $u=[u_{1},...,u_{m}]^{T}$includes the $m$smallest eigenvectors. Then, the measure of separability of subpopulations is defined as

. (C3)
