# Supplementary material for: Manifold Learning for Human Population Structure Studies
Source: PLoS One. 2012 Jan 17;7(1):e29901. doi: 10.1371/journal.pone.0029901 (PMC3260176; doi:10.1371/journal.pone.0029901)
Supplement: Appendix S11 — Proof D. (DOC) [file pone.0029901.s011.doc]

**Proof D**

Most of the work comes from the paper [14]. An undirected weighted graph can be described by $G=\{X,W\}$where $X$is a set of representation of the original data point $x_{i}$in the high dimensional space. Our goal is to seek minimizing

$\begin{matrix} F=\sum_{i=1}^{n} \sum_{j=1}^{n} ||y_{i}-y_{j}||^{2}w{}_{ij} \\ \begin{matrix} & =\sum_{i=1}^{n} (\sum_{j=1}^{n} w_{ij})||y_{i}||^{2} \end{matrix}+\sum_{j=1}^{n} (\sum_{i=1}^{n} w_{ij})||y_{j}||^{2}-2\sum_{i=1}^{n} \sum_{j=1}^{n} w_{ij}y_{i}^{T}y_{j} \\ \begin{matrix} & =Y^{T}(I-W)Y \end{matrix}, \end{matrix}$ (D1)

where $Y=[y_{1},...,y_{n}]^{T}$and $I$is an identity matrix.

PCA is to find project directions of the high dimensional data which have maximal variance:

$$\max\begin{matrix} & \end{matrix}a^{T}Ca$$

or we seek to remove the projection direction with minimal variance:

$\min\begin{matrix} & a^{T}Ca \end{matrix}$ (D2)

where . Let $Y=Xa$ and

$W=\frac{1}{n}11^{T}$. Then, problem (D2) can be written as

$\min\begin{matrix} & Y^{T}(I-W)Y \end{matrix}$. (D3)

We can see that PCA is the projection of the graph embedding with the intrinsic graph connecting all the nodes with equal weights$\frac{1}{n}$.

In the LLE, we define

$W'=W^{T}+W-W^{T}W$.

Then, the cost function in the LLE algorithm can be written as

$\min\begin{matrix} & Y^{T}(I-W')Y \end{matrix}$. (D4)

The equations (D3) and (D4) share the same formulation, but the graph embedding in the LLE has more complicated heterogeneous intrinsic structure than the graph embedding in PCA.
